# Genome-Wide Association Studies of Cognitive and Motor Progression in Parkinson’s Disease

**DOI:** 10.1002/mds.28342

**Published:** 2020-10-28

**Authors:** Manuela M.X. Tan, Michael A. Lawton, Edwin Jabbari, Regina H. Reynolds, Hirotaka Iwaki, Cornelis Blauwendraat, Sofia Kanavou, Miriam I. Pollard, Leon Hubbard, Naveed Malek, Katherine A. Grosset, Sarah L. Marrinan, Nin Bajaj, Roger A. Barker, David J. Burn, Catherine Bresner, Thomas Foltynie, Nicholas W. Wood, Caroline H. Williams-Gray, John Hardy, Michael A. Nalls, Andrew B. Singleton, Nigel M. Williams, Yoav Ben-Shlomo, Michele T.M. Hu, Donald G. Grosset, Maryam Shoai, Huw R. Morris

**Affiliations:** 1 Department of Clinical and Movement Neurosciences, Queen Square Institute of Neurology, University College London, London, UK; 2 UCL Movement Disorders Centre, University College London, London, UK; 3 Population Health Sciences, Bristol Medical School, University of Bristol, Bristol, UK; 4 Department of Neurodegenerative Diseases, Queen Square Institute of Neurology, University College London, London, UK; 5 Molecular Genetics Section, Laboratory of Neurogenetics, National Institute on Aging, National Institutes of Health, Bethesda, Maryland, USA; 6 Data Tecnica International, Glen Echo, Maryland, USA; 7 MRC Centre for Neuropsychiatric Genetics and Genomics, Institute of Psychological Medicine and Clinical Neurosciences, Cardiff University, Cardiff, UK; 8 Department of Neurology, Institute of Neurological Sciences, Queen Elizabeth University Hospital, Glasgow, UK; 9 Institute for Ageing and Health, Newcastle University, Newcastle Upon Tyne, UK; 10 Department of Clinical Neurosciences, University of Nottingham, Nottingham, UK; 11 Department of Clinical Neurosciences, John van Geest Centre for Brain Repair, University of Cambridge, Cambridge, UK; 12 Wellcome-MRC Cambridge Stem Cell Institute, University of Cambridge, Cambridge, UK; 13 Faculty of Medical Sciences, Newcastle University, Newcastle Upon Tyne. UK; 14 Reta Lila Weston Institute, UCL Queen Square Institute of Neurology, London, UK; 15 UK Dementia Research Institute, University College London, London, UK; 16 National Institute for Health Research (NIHR) University College London Hospitals Biomedical Research Centre, London, UK; 17 Institute for Advanced Study, The Hong Kong University of Science and Technology, Hong Kong, SAR, China; 18 Nuffield Department of Clinical Neurosciences, Division of Clinical Neurology, University of Oxford, Oxford, UK; 19 Oxford Parkinson’s Disease Centre, University of Oxford, Oxford, UK; 20 Department of Clinical Neurology, Oxford University Hospitals NHS Foundation Trust, Oxford, UK

**Keywords:** Parkinson’s disease, genetics, progression, genome-wide association study

## Abstract

**Background:**

There are currently no treatments that stop or slow the progression of Parkinson’s disease (PD). Case-control genome-wide association studies have identified variants associated with disease risk, but not progression. The objective of the current study was to identify genetic variants associated with PD progression.

**Methods:**

We analyzed 3 large longitudinal cohorts: Tracking Parkinson’s, Oxford Discovery, and the Parkinson’s Progression Markers Initiative. We included clinical data for 3364 patients with 12,144 observations (mean follow-up 4.2 years). We used a new method in PD, following a similar approach in Huntington’s disease, in which we combined multiple assessments using a principal components analysis to derive scores for composite, motor, and cognitive progression. These scores were analyzed in linear regression in genome-wide association studies. We also performed a targeted analysis of the 90 PD risk loci from the latest case-control meta-analysis.

**Results:**

There was no overlap between variants associated with PD risk, from case-control studies, and PD age at onset versus PD progression. The *APOE* ɛ4 tagging variant, rs429358, was significantly associated with composite and cognitive progression in PD. Conditional analysis revealed several independent signals in the *APOE* locus for cognitive progression. No single variants were associated with motor progression. However, in gene-based analysis, *ATP8B2*, a phospholipid transporter related to vesicle formation, was nominally associated with motor progression (*P* = 5.3 × 10 ^−6^).

**Conclusions:**

We provide early evidence that this new method in PD improves measurement of symptom progression. We show that the *APOE* ɛ4 allele drives progressive cognitive impairment in PD. Replication of this method and results in independent cohorts are needed.

Progression in Parkinson’s disease (PD) is heterogeneous, with some patients progressing rapidly, whereas others remain relatively stable over time.^1^ There is a clear need to identify genetic variants that affect symptom progression in PD. These genes and pathways could be targeted to develop therapies to stop or slow the progression of PD. Genetic factors could also help to stratify patients and predict progression more accurately in clinical trials.

Genome-wide association studies (GWASs) in PD have identified 90 independent loci associated with disease risk.^2^ However, the majority of PD GWASs have compared cases with healthy controls to identify variants linked to disease status. To identify variants that are associated with disease progression, it is necessary to compare phenotypes within patients.

Progression of clinical signs in PD can be measured in different ways,^3^ and there is no gold standard measure of progression, although the Movement Disorder Society Unified Parkinson’s Disease Rating Scale (MDS-UPDRS) part III and part II arc commonly used in clinical trials. Individual scales, including the MDS-UPDRS, are affected by measurement error, particularly for change over time,^4^ including rater subjectivity and practice effects in cognitive assessments. Therefore, combining multiple measures may improve the accuracy of measuring progression,^5,6^ as shown in the Huntington’s disease (HD) progression GWAS.^7^ In this study, we analyzed data from 3 large prospective longitudinal studies: Tracking Parkinson’s, Oxford Parkinson’s Disease Centre Discovery, and Parkinson’s Progression Markers Initiative (PPMI). We combined multiple measures of motor and cognitive progression using principal components analysis (PCA) to create progression scores. These scores were analyzed in GWASs to identify variants associated with composite (cross-domain), motor, and cognitive progression in PD.

## Methods

**Fig. 1.**
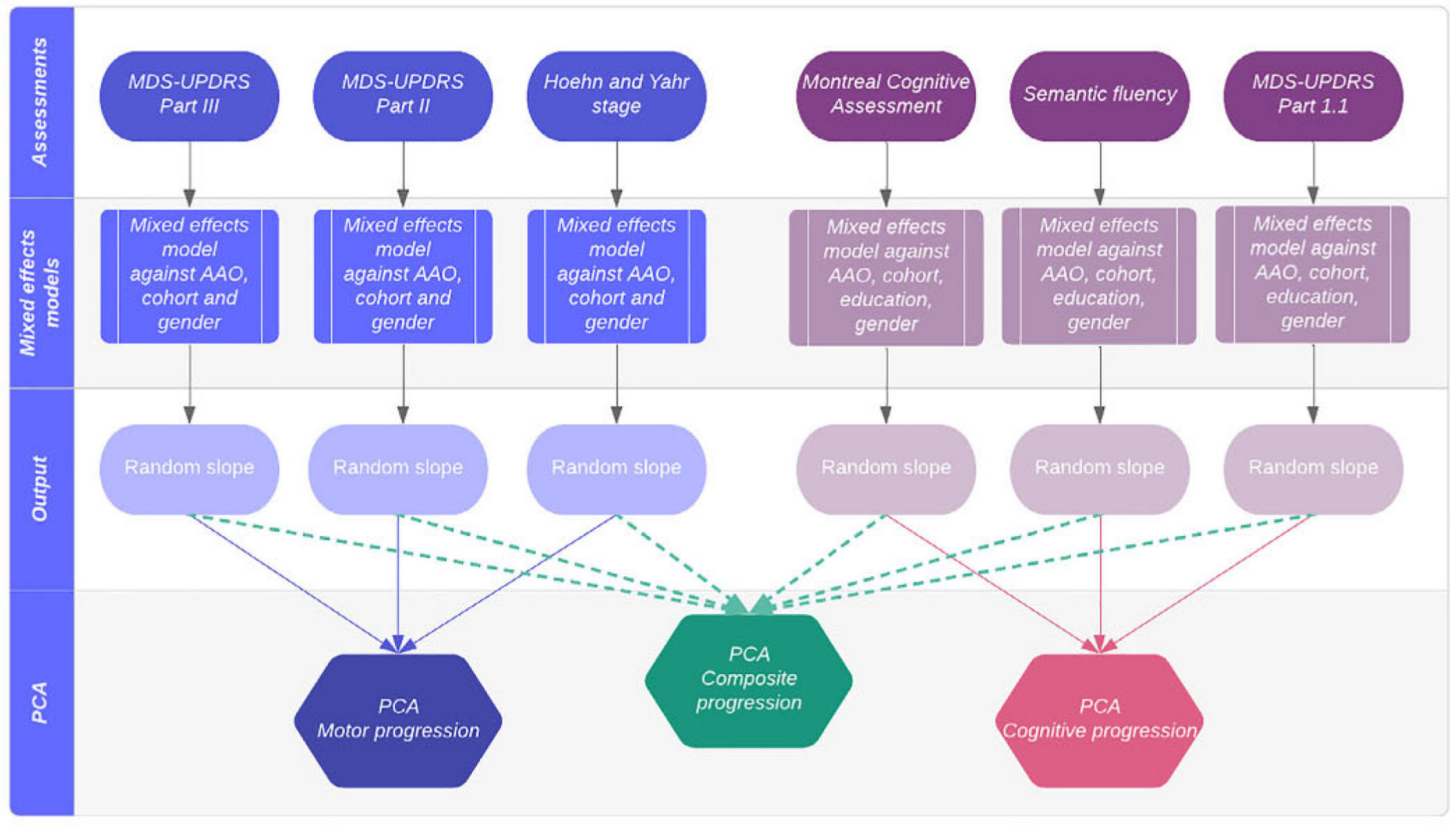
Steps to create composite, motor, and congnitive progression scores. AAO, age at onset. [Color figure can be viewed at wileyonlinelibrary.com]

**FIG. 2.**
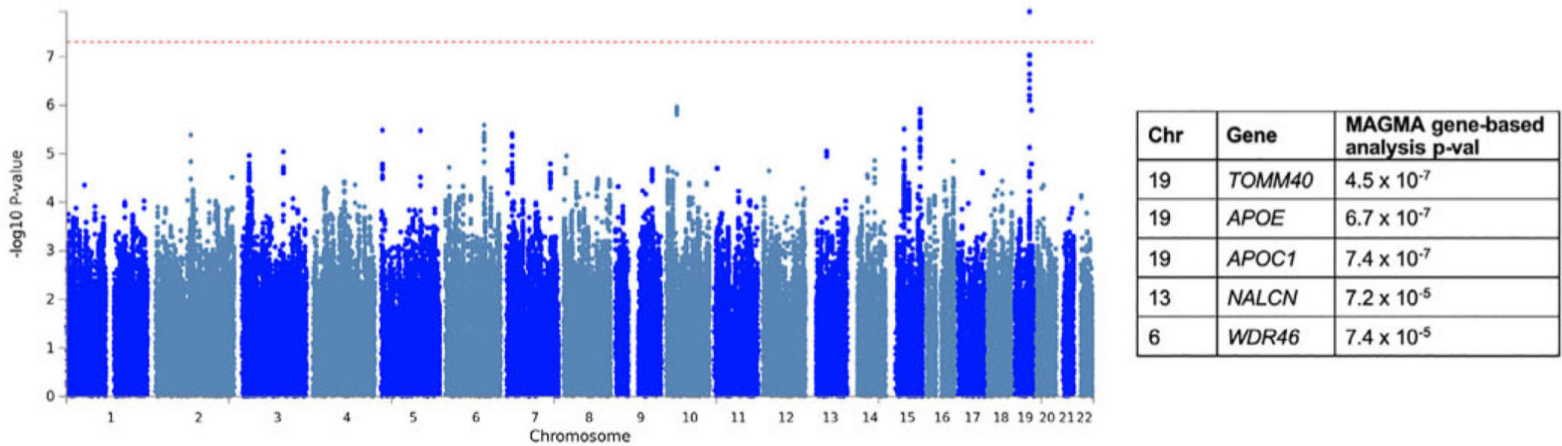
Manhattan plot for GWAS of composite progression. The red dashed line indicates the genome-wide significance threshold, *P* = 5×10^−8^. The top genes from the MAGMA gene-based analysis and *P* values are shown on the right. [Color figure can be viewed at wileyonlinelibrary.com]

Standard quality control procedures were performed in PLINK v1.9. The cohorts were gcnotyped, filtered, and imputed separately, but following the same quality control steps. Only variants with minor allele frequency > 1 % were included. The 3 data sets were merged after imputation, with only shared variants retained. Genetic principal components were generated and outliers removed (see Supplementary Methods and Figs. 1 and 2).

### Clinical Outcome Measures

Individual-level data from the cohorts were merged. To increase the power and the accuracy of the final progression scores, we performed all transformations and created progression scores from the merged data set as follows (Fig. 1).

Motor progression was assessed using MDS-UPDRS part III (clinician-assessed movement examination), MDS-UPDRS part II (patient-reported experiences of daily living), and Hochn and Yahr stage (clinician-assessed rating of impairment and disability).^8,9^ In PPMI, we used motor assessments conducted in the “off” medication state.

Cognitive progression was assessed using the Montreal Cognitive Assessment, semantic fluency, and item 1.1 of the MDS-UPDRS (cognitive impairment based on patient and/or caregiver report).

Raw scores were transformed into percentages and standardized to the population baseline mean and standard deviation within each cohort (Supplementary Methods).

### Analysis

#### Progression Scores

We derived severity scores from mixed-effects regression models using follow-up data up to 72 months. Each variable was regressed on age at onset, sex, cohort, and their interactions with time from disease onset. PD onset was based on participants’ self-reported symptom onset. For the cognitive measures, we included the number of years of education before higher education and whether higher education was undertaken as covariates. We included terms for subject random effects to account for individual heterogeneity in the intercept (baseline value) and slope (rate of progression).

We used random-effect slope values as the measure of “residual” progression not predicted by age at onset, cohort, sex, and education, for each individual. We performed PCA on these values after zero centering and scaling to have unit variance. The final progression scores from the PCA relate to the variability explained, and therefore the direction cannot be strictly interpreted. Patients who were missing clinical data (eg. MDS-UPDRS part III total) at all visits were not included in the PCA and subsequent GWAS analysis.

#### Removal of Non-PD Cases

Any patients who were diagnosed with a different condition during follow-up were removed from analyses. We also conducted sensitivity analyses to remove any eases that may have non-PD conditions but an alternative diagnosis had not yet been confirmed. First, we removed patients in Tracking Parkinson’s and Oxford Discovery who had a clinician-rated diagnostic certainty of PD < 90%.^10,11^ Second, we removed the fastest and slowest progressors in the top and bottom 5% of the distribution to address the possibility of confounding by misdiagnosis with more benign (eg, essential tremor) or more malignant (eg, multiple system atrophy) conditions.

#### GWAS

For each GWAS, we included the following covariates: cohort (to adjust for differences in genotyping data and measurement error) and the first 5 genetic principal components from the merged genotype data (to adjust for population substructure). GWASs were conducted in rvtests^12^ using the single-variant Wald test. Genome-wide complex trait analysis conditional and joint analysis (GCTA-COJO) was used to identify independent signals.^13,14^ Individuals carrying rare variants in *GBA, LRRK2,* or other PD genes were not excluded from the GWASs. We also performed sex-stratified analysis to identify if there are different genetic associations in men and women.

Genetic risk scores were calculated from the 90 loci from the PD case-control GWAS,^2^ and we analyzed the association with each progression score using linear regression.

#### GBA

We analyzed *GBA* rare variant carriers compared with noncarriers in a subset of patients, using Sanger sequencing data from Tracking Parkinson’s and whole-genome sequencing data from PPMI. In PPMI, only the following *GBA* variants were covered: N370S, T369M, E326K, and R463C. We classified patients as carrying a pathogenic *GBA* variant, including Gaucher’s disease variants and variants associated with PD but excluding novel variants, using previous studies.^15,16^ We analyzed *GBA* status in relation to the progression scores using linear regression, adjusting for cohort and the first 5 genetic principal components.

#### Levodopa-Equivalent Daily Dose-Adjusted Sensitivity Analyses

Medication may affect MDS-UPDRS part III scores, in particular in Tracking Parkinson’s and Oxford Discovery, in which patients were assessed in thc “on” state. To address this, we performed a sensitivity analysis adjusting for levodopa-equivalent daily dose (LEDD), as described in a previous study, in which we estimated the effect of levodopa on MDS-UPDRS part III scores^11^ (Supplementary Methods). Merely adjusting for treatment as a covariate is not adequate, as therapy is not a simple confounder but a direct outcome of the underlying symptom — individuals who have more severe symptoms are more likely to be treated^17^ and most likely with higher doses.

## Results

We included clinical data for 3364 PD patients with 12,144 observations (Table 1). Mean follow-up time ± SD was 4.2 ± 1.5 years, and mean disease duration at study entry was 2.9 ± 2.6 years. A total of 79.7% of patients had completed the 72-month follow-up visit.

Within the motor progression PCA, the first principal component explained 61.0% of the total variance. Within the cognitive domain PCA, the first principal component explained 59.8% of the total variance (Figs. S3–S6).

We found that the first principal components for motor and cognitive progression were moderately correlated (*r* = −0.35, *P* < 2.2 × 10^−16^; Table S1). We therefore conducted a PCA combining all motor and cognitive measures to create a composite progression score. The first principal component from this cross-domain PCA accounted for 41.0% of the joint variance (Figs. S7 and S8). Tables S2–S6 show how the raw scales and the motor, cognitive, and composite principal components are correlated. None of the principal components were associated with cohort (all *P*s > 0.9).

### GWAS of Composite Progression

After quality control, imputation, and merging, 5,918,868 variants were available for analysis. A total of 2755 PD patients had composite progression scores and passed genetic quality control. All GWAS lambdas were <1.05. One variant, rs429358, in chromosome 19 passed genome-wide significance (*P* = 1.2 × 10^−8^; Fig. 2, Table S7, Figs. S9 and S10). This variant tags the *APOE* ε4 allele. In the gene-based test, *APOE, TOMM40,* and *APOCA* reached significance (*P* < 2.8 × 10^−6^, correcting for the number of mapped protein coding genes). When we performed conditional analysis on the top single-nucleotide polymorphism (SNP), rs429358, there were no other SNPs that passed significance in this region (Fig. S11). The Reactome pathway cytosolic sulfonation of the small-molecule pathway was significantly enriched (*P* = 6.9 × 10^−6^ ).

### GWAS of Motor Progression

**FIG. 3.**
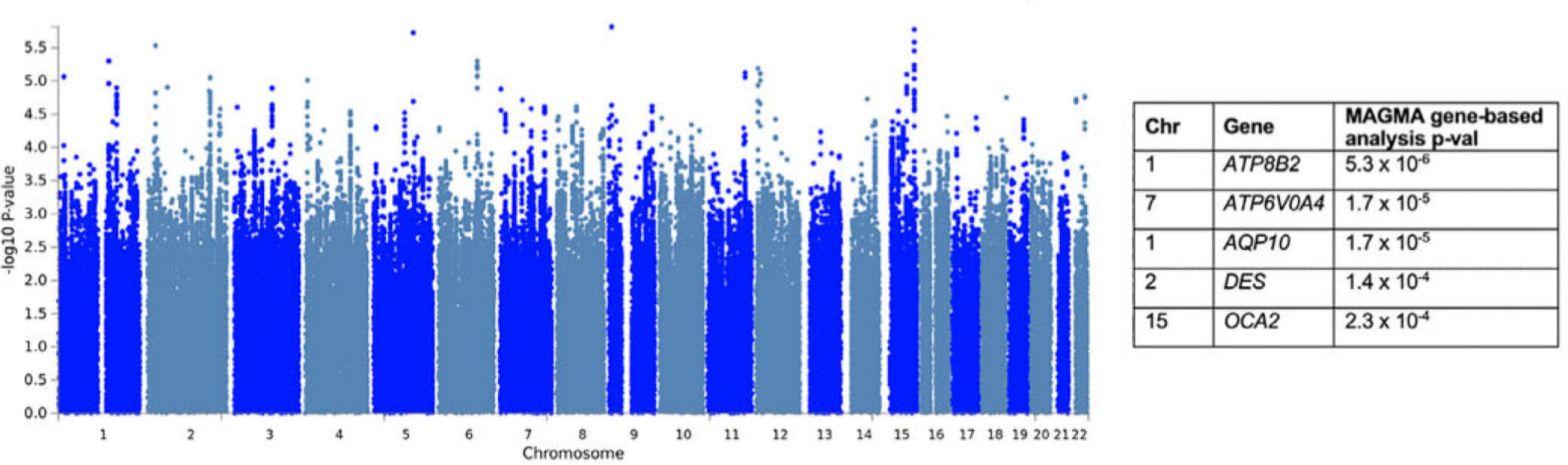
Manhattan plot for the GWAS of motor progression. Genome-wide significance is the standard *P* = 5 × 10^−8^ (not indicated in the figure). The top genes from the MAGMA gene-based analysis and *P* values are shown on the right. [Color figure can be viewed at wileyonlinelibrary.com]

A total of 2848 PD patients had motor progression scores and genotype data. No variants passed genome-wide significance (Fig. 3, Table S8). However, in the gene-based test, *ATP8B2* in chromosome 1 was associated with motor progression (*P* = 5.3 × 10^−6^; Figs. S12 and S13), although this did not reach significance correcting for the number of mapped genes (*P* = 2.81 × 10^−6^).

We conducted follow-up GWASs in each cohort separately (Table S9) and each motor scale separately (without combining in PCA) to confirm that the results were not driven by a single cohort or a single scale. These results show that associations are strengthened with the PCA approach (Table S10).

Our top variant in chromosome 1, rs35950207, was associated with motor progression, *P* = 5.0 × 10 ^−6^. We examined the associations for this SNP in the previous progression GWAS^18^ (https://pdgenetics.shinyapps.io/pdprogmctagwasbrowser/); rs35950207 was not significantly associated with binomial analysis of Hoehn and Yahr stage 3 or more at baseline (beta = 0.27, *P* = 0.03).

The variant rs35950207 is 2 kb upstream of *AQP10*. It is an expression quantitative trait loci (eQTL) for *AQP10* in whole blood (GTEx, *P* = 1.7 × 10^−6^; eQTLGen, *P* = 3.62 × 10^−139^) and other tissues (subcutaneous adipose, skin, esophagus, testis, and heart). It is also an eQTL for *ATP8B2* in blood (GTEx, *P* = 1.5 × 10^−5^; eQTLGen, *P* = 7.84 × 10^−42^) and in the cerebellum (GTEx, *P* = 7.8 × 10^−5^). *GBA* is also located in chromosome 1, and *GBA* variants are associated with both PD risk and progression.^19^ However, rs35950207 is not in linkage disequilibrium with any of the main *GBA* variants that are implicated in PD (p.E326K, p.N370S, p.L444P, p.T369M).

In chromosome 5, the top SNP in the variant-based analysis was rs17367669, but there were no genes in this region that approached significance in the gene-based analysis. This variant is closest to *LOC100505841,* zinc finger protein 474-like gene. No significant eQTLs were identified for this variant.

### GWAS of Cognitive Progression

**FIG. 4.**
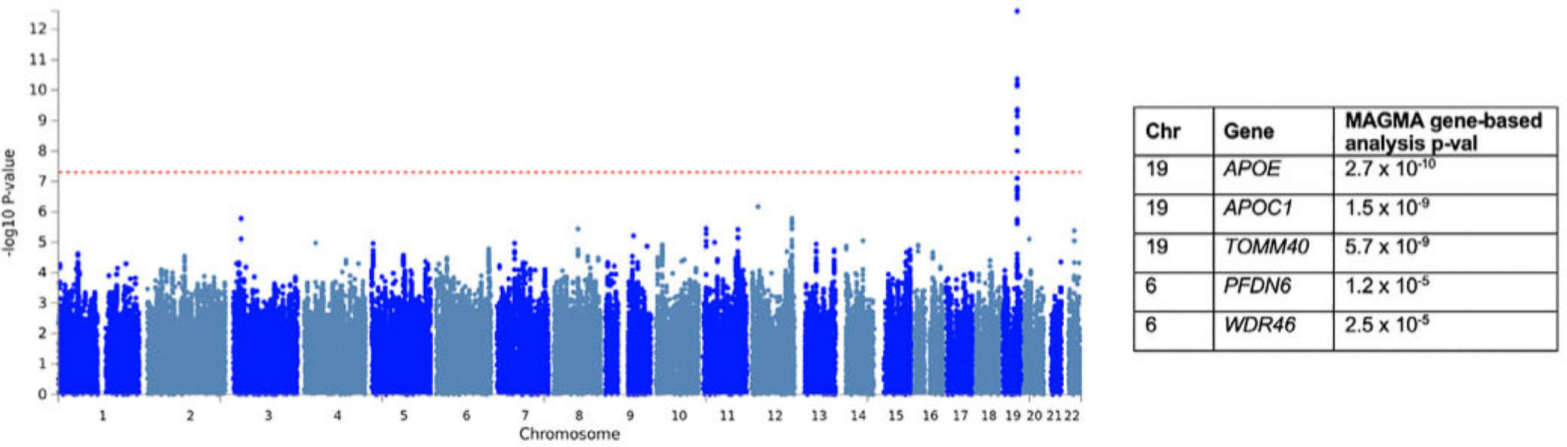
Manhattan plot for the variant-based GWAS of cognitive progression. The red dashed line indicates the genome-wide significance threshold, *P* = 5×10^−8^. The top genes from the MAGMA gene-based analysis and *P* values are shown on the right. [Color figure can be viewed at wileyonlinelibrary.com]

A total of 2788 patients had cognitive progression scores and genotype data. The top variant was rs429358, which tags the *APOE* ε4 allele (*P* = 2.53 × 10^−13^; Fig. 4, Table S11, Figs. S14 and S15). Figure S16 shows that ε4 carriers had more severe cognitive progression. *APOE* was also significantly associated with cognitive progression in the gene-based analysis, in addition to *APOC1* and *TOMM40.* Follow-up analyses showed that the effects for the top 5 independent SNPs were consistent in each cohort and each scale (Tables S12 and S13).

When we performed conditional analysis on the top SNP, rs429358, a group of SNPs still passed genome-wide significance, indicating independent signals (Fig. S17). The top SNP was rs6857 (beta = −0.33, P = 4.4 × 10^−11^). This is a 3’ UTR variant in *NECTIN2.* We also conditioned on the other *APOE* SNP, rs7412, in addition to rs429358 (if both rs429358 and rs7412 harbor the C alleles, then this codes the ε4 allele). This did not change the results.

When conditioning on both rs429358 and rs6857, there were still several SNPs that passed significance, the top being rs12721051, an intronic variant in *APOC1.*

We found frequencies of *APOE* genotypes similar to those of previous studies^20^ (Table S14).

#### LEDD-Adjusted Analyses

When we performed GWASs of composite progression and motor progression after adjusting for LEDD, we did not find substantial differences. No SNPs passed genome-wide significance. The top SNP for composite progression was still rs429358, and this was in the same direction and similar effect size as in the main analysis (beta = 0.33, *P* = 8.8 × 10^−8^). For motor progression, the top SNP was also the same as in the main analysis and *ATP8B2* and *AQP10* still the top genes in the MAGMA gene analysis, although not genome-wide significant.

#### Sex-Stratified Analyses

The *APOE* locus passed genome-wide significance only in men for composite progression and cognitive progression (*P* < 5 × 10^−8^). Other than this locus, there were no SNPs that passed significance. These analyses arc underpowered, and sex differences need to be investigated in more detail.

#### Targeted Assessment of PD Risk Loci

Of the 90 risk variants from the PD case-control GWAS,^2^ 73 were present in our final data set, including the *SNCA* and *TMEM17S/GAK* variants associated with PD age at onset.^21^ No variants passed analysis-wide significance *(P =* 0.05/73). Variants with at least 1 association, *P* < 0.05, are shown in Figure S18.

We found that only a small number of risk variants were associated with progression, with *P* < 0.05. The variant rs35749011 was associated with both composite progression (beta = 0.40, *P* = 0.003) and cognitive progression (beta = −0.37, *P* = 0.002), but not motor progression (beta = 0.20, *P* = 0.09). This variant is in linkage disequilibrium with the *GBA* p.E326K variant (also known as p.E365K), D’ = 0.90, *R*^2^ = 0.78.

We also extracted results for other candidate variants that have been implicated in PD progression (Fig. S19). We did not find that the top variant, rs382940, in *SLC44A1* that was associated in progression to Flochn and Yahr stage 3 from the Iwaki GWAS^18^ was associated with either composite, motor, or cognitive progression in our GWASs.

Overall, we did not find any overlap between the variants associated with PD risk, age at onset, and progression. Our Linkage Disequilibrium Score Regression (LDSC) results also suggested very little overlap between each of the progression GWASs and PD case-control GWAS (all *P*s > 0.5).

#### PD Genetic Risk Score

A total of 73 PD risk SNPs were present in our genotype data, and 2 proxies were identified for missing variants (Table S15). The risk score was nominally associated with cognitive progression (beta = −0.098, *P* = 0.04) but not composite (beta = 0.09, p=0.12) or motor progression (beta = 0.02, *P* = 0.69).

### GBA

*GBA* data was available for 2020 patients from Tracking Parkinson’s and PPMI. 194 (9.6%) carried a pathogenic variant in *GBA* (Table S16). *GBA* status was significantly associated with composite progression (beta = 0.40, *P* = 0.001) and cognitive progression (beta = −0.35, *P* = 0.0008), but not motor progression (beta = 0.18, *P* = 0.10).

### Removal of Potential Non-PD Cases

Removing patients with <90% diagnostic certainty did not substantially affect our results; the top signals had slightly weaker associations in these sensitivity analyses. When we removed the extreme 5% of progressors, the top results from the main GWASs had the larger *P* values, although the direction of effects were the same (Tables S17 and S18).

## Discussion

We used a new method of analyzing clinical progression in PD by combining multiple assessments in a data-driven PCA to derive scores of composite, motor, and cognitive progression in large clinical cohorts.

Our study contributes to evidence that improving the phenotypic measure can increase power in genetic studies. We showed that associations at the top signals strengthened when using the combined motor and cognitive progression scores compared with using the scales separately. The HD progression GWAS also showed that motor, cognitive, and brain imaging measures were well correlated and successfully identified a variant in *MSH3* associated with composite progression.^7^ Other studies show prediction accuracy of PD status or progression (such as development of cognitive impairment) is improved by combining multiple clinical, genetic, and biomarker factors.^6,22^

In PD, there are many different scales for assessing symptoms. Each scale has a degree of measurement error^4^ and different sensitivity to progression of underlying symptoms.^23^ PCA is a data-driven approach that combines multiple measures to identify latent components that explain the most variability in the data, and these may more accurately reflect disease progression.

Our progression GWASs have 2 main findings. First, we replicated previous findings for *APOE* ε4. Many studies have shown that the ɛ4 allele is associated with dementia in PD,^20,24–26^ and potentially separately from the risk of Alzheimer’s disease (AD).^27^ One possible mechanism is that *APOE* is associated with amyloid-β pathology, as comorbid AD pathology is common in PD patients with dementia (PDD) at postmortem.^28^ Alternatively, *APOE* may drive cognitive decline independently of amyloid/AD pathology. Recent animal model work has shown that the ɛ4 allele is independently associated with α-synuclcin pathology and toxicity.^29^ In addition, the ɛ4 allele is overrepresented in dementia with Lewy body cases with “pure” Lewy body pathology, compared with PDD cases.^30^ A systematic review showed that limbic and neocortical α-synuclcin pathology had the strongest association with PD dementia.^28^ Further work is needed to determine the mechanisms by which *APOE* influences cognitive decline.

In the *APOE* locus, there may be multiple independent signals for cognitive progression. This is similar to AD, in which multiple risk loci have been located in chromosome 19 in addition to *APOE,* including *TOMM40, APOC1,* and more distant genes. This study was not powered to conduct analyses stratified by *APOE* genotype, as has been done in AD. ^31^ Further work is needed to fine-map this region and determine if there are other genes that contribute to cognitive progression.

We identified a novel signal in *ATP8B2* associated with motor progression in a gene-based analysis. This gene encodes an ATPase phospholipid transporter (type 8B, member 2). Phospholipid translocation may be important in the formation of transport vesicles.^32^ This gene has not been reported in PD or other diseases and needs to be tested in other cohorts.

Our sensitivity analysis adjusting for LEDD suggests that levodopa may influence the absolute scores in the MDS-UPDRS part III but docs not influence the rate of progression, and this was shown in a previous study.^33^ We also found that the mean rate of change in MDS-UPDRS part III was comparable in Tracking Parkinson’s/Oxford Discovery and PPMI (Table 1), despite the different medication states. Together, these suggest that medication has not influenced our results for motor progression.

We have shown that the genetics of PD risk and progression are largely separate. In our targeted analysis of PD risk variants, *GBA* p.E326K was nominally associated with composite and cognitive progression. Analysis of sequencing data showed that *GBA* status was strongly associated with composite and cognitive progression, but not motor progression. Previous studies show that *GBA* variants arc associated with rapid progression and mortality^34–39^; however, many of these studies have longer follow-up or patients with longer disease duration. This may explain why we did not find a strong effect for motor progression and is supported by analysis of *GBA* in patients at an earlier stage of the disease.^15^ In addition, previous studies have used different methods to measure progression. Our unbiased genome-wide search suggests that, in addition to *GBA,* there are potentially other genes that are important for PD progression.

Our targeted analysis showed that only a few PD risk variants were nominally associated with progression, similar to thc previous PD progression GWAS.^18,40^ This suggests that there is minimal overlap in the genetic architecture of PD risk and PD progression. Similarly, the age at onset GWAS showed only a partial overlap with the genetics of PD risk.^21^ We now have the ability to study progression through the integration of detailed clinical data with genome-wide genetic variation in large-scale studies, and this can improve our understanding of the biology of progression.

We did not replicate the finding for the *SLC44A1* variant that was associated with progression to Hoehn and Yahr stage 3 in a previous PD progression GWAS.^18^ We have used different methods and a different phenotype to analyze PD progression. Further progression GWASs are needed to replicate both sets of results, and other metrics for PD progression could be analyzed, such as mortality.

Although no other large genome-wide GWASs have investigated PD progression, many candidate gene studies have nominated common genetic factors associated with progression. Aside from *APOE,* common variants in MAPT,^1,41–43^ *COMT,*^24,42^ *BDNE, MTHER,* and *SORL1*^44^ have been reported to influence cognitive decline (reviewed in Fagan and Pihlstrom^45^). For motor progression, other than *GBA,* common variants in *SNCA* have been suggested to influence the rate of decline, although these studies are small and have not been confirmed in large studies.^26,46–49^ A small GWAS of motor and cognitive progression identified suggestive loci in *C8orf4* and *CLRN3*,^50^ although these have not been replicated. A novel machine-learning approach found that variation in *LING02* was associated with change in the MDS-UPDRS,^51^ although again this finding needs independent replication. We did not replicate these findings, possibly because we were underpowered as a GWAS to detect variants with smaller effects or because we have analyzed progression using different methods. However, many of these previous studies are small, and some associations have not been convincingly replicated.

Our study has some limitations. Follow-up was limited to 72 months, and longer follow-up is needed to detect variants that may influence progression in later disease stages, such as *GBA*.

We may also be underpowered to detect variants with smaller effects on progression. Although the FID GWAS identified significant signals in smaller samples,^7^ analysis of PD progression is more complex because of slower progression, greater heterogeneity in genetic risk and rate of progression between patients, and greater dissociation between motor and cognitive progression. Our findings need to be tested in independent cohorts, and the lack of independent replication is another limitation of this study.

A third limitation is that symptom progression may be influenced by non-SNP variants (such as rare variants or structural variants) and gene—gene interactions that would be missed by GWASs, or environmental factors and comorbidities.

A final limitation is the potential inclusion of patients that have non-PD conditions. We did not find that our results changed substantially when we excluded patients with diagnostic certainty < 90%. However, certainty data were not available for PPMI, and abnormal dopamine transporter scans cannot differentiate between PD and other degenerative parkinsonian conditions.^52^ Despite this, our sensitivity analysis suggest that our results are not being driven by non-PD conditions. Our GWASs also did not identify loci that arc associated with PSP risk, including *MAPT, MOBP*,^53^ or rs2242367 near *LRRK2* associated with PSP progression.^54^

Many of our top variants had weaker signals when we excluded the fastest-and slowest-progressing patients. With our duration of follow-up, we should have excluded the majority of non-PD patients, as diagnostic accuracy improves after 5-year duration of disease^1,55^; however, it is possible that some have not been excluded. Analysis of pathologically confirmed PD cases is needed to resolve this issue. Alternatively, this may indicate that genotypes have different effects in the most extreme progressors. This could be because of comorbidities such as vascular burden^56^ or interactions between synuclein and copathologies (such as amyloid, and tau)^57,58^ in the rapid progressors that exacerbates clinical progression.

This study is the first to use a PCA data reduction method to assess PD progression, based on a successful approach in HD. We robustly replicated the association between *APOE* ɛ4 and cognitive progression and have identified other genes that may be important. These advances are essential to understanding the biology of disease progression and nominating therapeutic targets to stop or slow PD progression.

## Supplementary Material

Supplementary Material

## Figures and Tables

**Table 1.** Cohort demographics at baseline

Demographics at baseline	Tracking Parkinson’s	Oxford Discovery	PPMI	ALL

Number of PD patients	1966	985	413	3364
Total number of visits analyzed	5936	3142	3066	12.144
Mean length of follow-up (years)	3.8 (1.4)	4.3 (1.7)	5.4 (1.2)	4.2 (1.5)
Male (%)	65.2%	64.2%	65.4%	65.0%
Age at onset (years)	64.4 (9.8)	64.5 (9.8)	59.5 (10.0)	63.9 (10.0)
Age at diagnosis (years)	66.3 (9.3)	66.1 (9.6)	61.0(9.7)	65.6 (9.6)
Age at study entry (years)	67.6 (9.3)	67.4 (9.6)	61.5(9.8)	66.8 (9.7)
Disease duration — time from symptom onset to assessment (years)	3.2 (3.0)	2.9 (1.9)	2.0 (2.0)	2.9 (2.6)
Time from diagnosis to assessment (years)	1.3 (0.9)	1.3 (0.9)	0.5 (0.5)	1.2 (0.9)
MDS-UPDRS part III	22.9 (12.3)	26.8 (11.1)	20.7 (8.8)	23.8 (11.7)
MDS-UPDRS part III annual change^a^	1.9 (3.7)	2.1 (3.5)	1.8 (2.2)	2.1 (6.2)
MDS-UPDRS part II	9.9 (6.6)	8.9 (6.2)	5.8 (4.1)	9.0 (6.3)
MDS-UPDRS part II annual change^a^	1.3 (1.6)	1.3 (1.6)	0.9 (1.1)	1.3 (2.8)
Hoehn and Yahr stage mean^b^	1.8 (0.6)	1.9 (0.6)	1.6 (0.5)	1.8 (0.6)
Hoehn and Yahr stage annual change	0.1 (0.2)	0.06 (0.1)	0.08 (0.1)	0.06 (0.3)
Hoehn and Yahr stage 0 to 1.5 (%)	48.1%	23.2%	44.8%	40.4%
Hoehn and Yahr stage 2 to 2.5 (%)	45.1%	68.8%	54.7%	53.2%
Hoehn and Yahr stage 3^c^ (%)	6.8%	8.1%	0.5%	6.4%
MoCA total (adjusted for education)	24.9 (3.6)	24.5 (3.5)	27.1 (2.3)	25.0 (3.6)
MoCA total annual change	−0.1 (0.9)	−0.1 (0.8)	−0.2 (0.6)	−0.1 (1.5)
Semantic fluency^c^	21.8 (6.9)	34.7 (9.0)	21.0 (5.4)	25.5 (9.5)
Semantic fluency annual change	−0.2 (1.5)	−0.5 (2.0)	−0.1 (0.9)	−0.5 (3.0)
MDS-UPDRS part I.1	0.5 (0.7)	0.5 (0.6)	0.3 (0.5)	0.5 (0.7)
MDS-UPDRS part I.1 annual change	0.07 (0.2)	0.05 (0.2)	0.07 (0.1)	0.05 (0.3)

SD, standard deviation; PPMI, Parkinson’s Progression Markers Initiative; PD, Parkinson’s disease: MDS-UPDRS, Movement Disorder Society Unified Parkinson’s Disease Rating Scale; MoCA, Montreal Cognitive Assessment. Mean (SD) shown unless otherwise indicated.

a Annual change score derived from a mixed-effects model of the raw scores as a function of years from onset, with subject random effects to account for individual heterogeneity in the intercept (baseline values) and slope (rate of progression). No other covariates were included in the model within each cohort. For the overall value, we adjusted for cohort and the interaction between cohort and years from onset.

b Tracking Parkinson’s used the modified Hoehn and Yahr stage scale, whereas Oxford Discovery and PPMI used the original scale. Hoehn and Yahr stage proportions are shown as a total of the number of people with nonmissing Hoehn and Yahr ratings at baseline.

c Instructions and timing for the semantic fluency task were slightly different between cohorts (completed within 60 or 90 seconds). To account for these differences, we standardized all scales within each cohort separately (see Methods section).
