# Supplementary material for: Genome-Wide Association Studies of Cognitive and Motor Progression in Parkinson’s Disease
Source: Mov Disord. Author manuscript; Available in PMC 2022 Apr 29. (PMC9053517; doi:10.1002/mds.28342)
Supplement: Supplementary Material [file NIHMS1788954-supplement-Supplementary_Material.pdf]

SUPPLEMENTARY MATERIAL: Genome-wide association studies of cognitive and motor progression in Parkinson's disease

Table of Contents

Supplementary Methods....................3

References....................33

Supplementary Figures

Supplementary Figure 1. First two genetic principal components plotted for each cohort, from the final genetic PCA (after removing outliers in two passes). 6

Supplementary Figure 2. First two principal components, generated from a PCA merged with HapMap data. 6

Supplementary Figure 3. Scree plot and plot showing the proportion of variance explained in the motor progression principal component analysis. 7

Supplementary Figure 4. Correlation matrix between the random slopes for the motor progression variables. 7

Supplementary Figure 5. Scree plot and plot showing the proportion of variance explained in the cognitive progression principal component analysis. 8

Supplementary Figure 6.Correlation matrix between the random slopes for the cognitive progression variables. 8

Supplementary Figure 7. Scree plot and plot showing the proportion of variance explained in the composite (cross-domain - both motor and cognitive variables) progression principal component analysis....................9

Supplementary Figure 8. Density plot showing the distribution of the composite progression scores, with the normal distribution curve shown in red. 9

Supplementary Figure 9. Composite progression GWAS: Manhattan plot for the gene-based test from MAGMA. 10

Supplementary Figure 10. QQ plots for the GWAS of composite progression (variant-based and gene-based tests)....................10

Supplementary Figure 11. Regional association plots of the Chromosome 19 locus associated with composite progression. 11

Supplementary Figure 12. Manhattan plot of the gene-based test for motor progression from MAGMA. 12

Supplementary Figure 13.QQ plots for the GWAS of motor progression (variant-based and gene-based tests)....................12

Supplementary Figure 14.Manhattan plot of the gene-based test for cognitive progression from MAGMA....................13

Supplementary Figure 15. QQ plots for the GWAS of cognitive progression (variant-based and gene-based tests)....................13

Supplementary Figure 16. Raw MoCA scores in each cohort by *APOE* ε4 status (carriers vs. non-carriers). 14

Supplementary Figure 17. Regional association plots of the Chromosome 19 locus associated with cognitive progression. 15

Supplementary Figure 18. Forest plots for the top SNPs for motor and cognitive progression, showing effect sizes of the top SNPs in different cohorts. 18

Supplementary Figure 19. Heatmap of the PD GWAS risk loci and their association with composite, motor, or cognitive progression. 19

Supplementary Figure 20. Heatmap of candidate variants and their association with composite, motor, or cognitive progression. 20

Supplementary Tables

Supplementary Table 1. Correlation between motor progression principal components and cognitive progression principal components. 21

Supplementary Table 2. Correlation between the principal components from combined progression PCA and random slopes from individual measures. 21

Supplementary Table 3. Outputs from mixed effects models showing the relationship between the longitudinal change in the raw scales (before percentage transformation and standardisation) and the composite progression score, in each cohort separately. 21

Supplementary Table 4. Correlations between the composite progression components and the motor and cognitive components. 22

Supplementary Table 5. Correlations between the motor principal components and the random slopes from the individual motor measures. 22

Supplementary Table 6. Correlations between the cognitive principal components and the random slopes from the individual motor measures. 22

Supplementary Table 7. Top 10 independent SNPs from the GWAS of composite progression. 23

Supplementary Table 8. Top 10 independent SNPs from the GWAS of motor progression. 24

Supplementary Table 9. Motor progression GWAS performed in each cohort separately. ...25

Supplementary Table 10. Motor progression GWAS performed for each scale separately. .25

Supplementary Table 11. Top 10 independent SNPs from the GWAS of cognitive progression. 26

Supplementary Table 12. Cognitive progression GWAS performed in each cohort separately. 27

Supplementary Table 13. Cognitive progression GWAS performed for each scale separately. 27

Supplementary Table 14. APOE genotype frequencies. 28

Supplementary Table 15. PD risk SNPs missing for calculation of the genetic risk score, and whether a proxy was identified. 29

Supplementary Table 16. GBA variants included as pathogenic, and their frequencies. 30

Supplementary Table 17. Sensitivity analysis excluding PD cases with less than 90% diagnostic certainty. 31

Supplementary Table 18. Sensitivity analysis excluding fastest and slowest progressing cases (top and bottom 5% of each distribution)..32

Supplementary Methods

Cohorts

Tracking Parkinson's is an observational, UK multi-centre study[1]. Participants with a clinical diagnosis of PD were recruited between 2012 and 2014. Standardised clinical assessments were conducted every 1.5 years.

Oxford Discovery is another UK observational, multi-centre study[2]. The inclusion criteria were the same as in Tracking Parkinson's, and almost the same assessments were collected every 1.5 years.

PPMI (http://www.ppmi-info.org/) is a multi-centre study of newly diagnosed PD patients[3]. PPMI data was downloaded on 14/08/2019. Only data from the annual visits was analysed from PPMI, as the motor assessments were performed in the technically defined 'off' state at these visits.

All studies used the same Queen Square Brain Bank diagnostic criteria for PD. Patients who received alternative diagnoses during follow-up or had neuroimaging results conflicting with a PD diagnosis were excluded from analyses. All studies were conducted according to good clinical practice and received approvals from local ethics committees, and patients provided informed consent.

Clinical outcome measures

The motor and cognitive measures were chosen prior to the analysis. Only assessments conducted in all cohorts were included. We selected measures shown to rate motor and cognitive function semi-objectively, in an attempt to minimise observer bias. We did not include scales which may be affected by a combination of motor, cognitive, and other non-motor symptoms.

To ensure the different measures were comparable, we first converted raw scores into a percentage of the maximum score for that scale, with higher scores indicating worse symptoms. The MoCA was reverse scored to count the number of incorrect items out of the maximum score of 30. Semantic fluency was reverse scored out of the highest individual score at baseline in each cohort.

Each measure was normalised to the population baseline mean and standard deviation within each cohort, to adjust for any differences in the scales or task instructions between cohorts.

This also ensures that the measures are on the same scale, and preserves data on longitudinal change.

Genotyping and quality control

DNA samples from Tracking Parkinson's were genotyped using the Illumina HumanCore Exome array with custom content[1]. Samples from Oxford Discovery were genotyped on the Illumina HumanCore Exome-12v1.1 or the Illumina InfiniumCore Exome-24 v1.1 arrays. For PPMI, whole-genome sequencing data was used (see https://www.ppmi-info.org/). Only variants that passed filters in the joint calling process were included.

Prior to imputation, individuals with low overall genotyping rates (<98%), related individuals (Identity-By-Descent PIHAT>0.1), and heterozygosity outliers (>2SDs away from the mean) were removed, as were individuals whose clinically reported biological sex did not match genetically determined sex.

PCA was conducted on a linkage disequilibrium (LD) pruned set of variants after merging with European samples from the HapMap reference panel. Individuals who were >6SDs away from the mean of any of the first 10 principal components were removed.

Variants were removed if they had a low genotyping rate (<99%), Hardy-Weinberg Equilibrium p-value $< 1 \times 10^{-5}$ and minor allele frequency < 1%.

Following quality control, genotypes for Tracking Parkinson's and Oxford Discovery were imputed separately to the 1,000 Genomes Project reference panel (phase 3 release 5)[4] using the Michigan Imputation Server (https://imputationserver.sph.umich.edu). Only variants with imputation quality >0.8 were retained, to keep only high-quality calls to merge across the cohorts. Tracking Parkinson's and Oxford Discovery data was lifted over to genome build hg38 using liftOver (https://genome.ucsc.edu/cgi-bin/hgLiftOver) to merge with PPMI. PPMI data was not imputed as this was whole-genome sequencing data.

Following imputation and merging, twenty genetic principal components were generated from a linkage-pruned SNP set (removing SNPs with an r^2>0.02 in a 1000kb sliding window shifting 10 SNPs at a time). The first 2 components were plotted to check that there were no differences between the cohorts. We removed extreme outliers from the first 5 principal components (>6SDs away from the mean). The genetic principal components were then recalculated after removing outliers. These first 5 new principal components were included as covariates in the GWAS to adjust for population substructure. Additional outliers who were >6SDs away from the mean of any of the first 5 principal components were excluded.

GWAS and follow-up analyses

Functional Mapping and Annotation of GWAS (FUMA; https://fuma.ctglab.nl/) was used with standard settings to annotate, prioritise, and visualize GWAS results[5]. Gene-based and gene-set analyses were conducted in FUMA with Multi-marker Analysis of GenoMic Annotation (MAGMA). MAGMA maps SNPs to genes and then tests the association between the genes and phenotype (gene-based analysis)[6]. We looked for enrichment of gene-sets or pathways in Gene Ontology (GO; MsigDB c5), Reactome, and Kyoto Encyclopedia of Genes and Genomes (KEGG). GTEx (https://gtexportal.org/) and the eQTLGen Consortium (http://www.eqtlgen.org/index.html) were used to look up expression quantitative trait loci (eQTLs). LDlink (https://ldlink.nci.nih.gov/) was used to calculate linkage between SNP pairs (using LDpair) in European populations excluding the Finnish population.

Clinical data was managed and cleaned using STATA (version 15.1, StataCorp, Texas, USA). Other statistical analysis was conducted in R v3.4.1[16].

For the PD Genetic Risk Scores, if SNPs were missing in our genotype data, proxies were identified using LDproxy if $r^2>0.9$. The association between the genetic risk score and each progression score was assessed using linear regression, adjusting for cohort and the first 5 genetic principal components. LD Score regression (LDSC)[7,8] was used to estimate the genetic correlation between the progression GWASs and the PD case-control GWAS using summary statistics excluding 23andMe samples[9].

Sex-stratified analysis

We standardised variables and created the clinical progression scores separately by sex and analysed these in GWASs separately.

Levodopa-equivalent Daily Dose (LEDD)-adjusted sensitivity analyses

Using the recommended method in previous studies[10], we added a sensible constant to the MDS-UPDRSIII scores to estimate what they would be if the patients were untreated, according to LEDD at each timepoint. We used data from the ELLDOPA study (personal communications)[11]. First, we converted UPDRS values from the ELLDOPA study to the MDS-UPDRS equivalent differences[12]. Second, we used a square root regression model at each timepoint to estimate the effect of different levodopa doses on the MDS-UPDRSIII[13]. This was only performed as a sensitivity analyses, as it involves extrapolation and the range of LEDD in our study exceeds that from the ELLDOPA data.

Supplementary Figure 1. First two genetic principal components plotted for each cohort, from the final genetic PCA (after removing outliers in two passes).

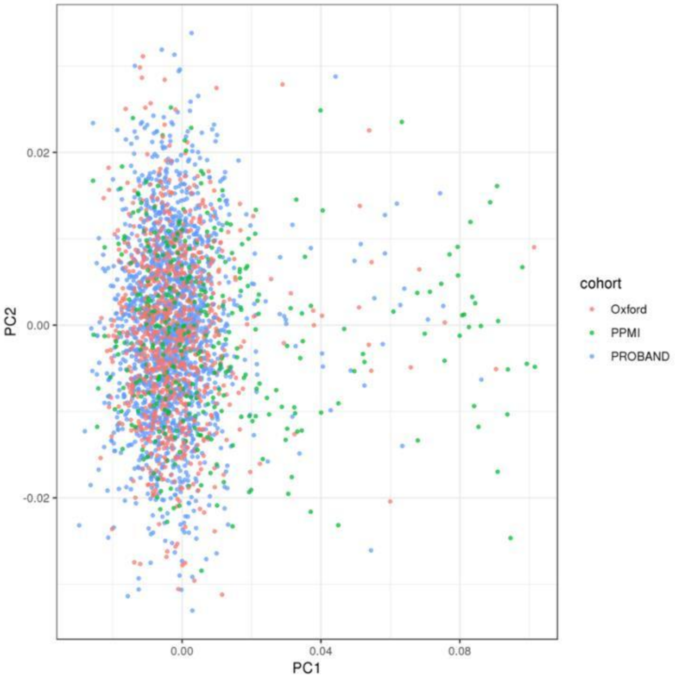

Supplementary Figure 2. First two principal components, generated from a PCA merged with HapMap data.

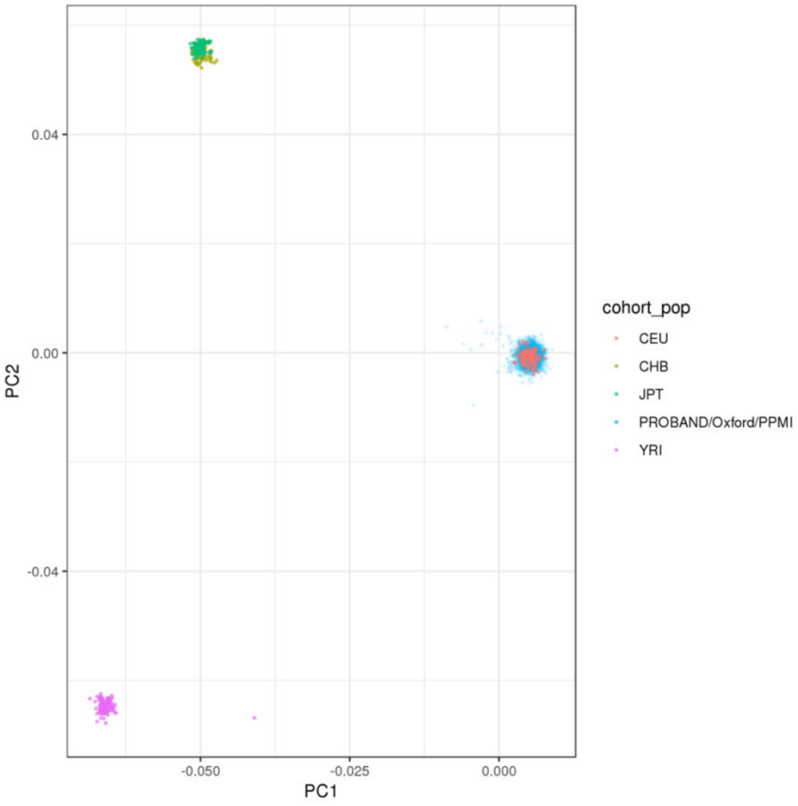

Supplementary Figure 3. Scree plot and plot showing the proportion of variance explained in the motor progression principal component analysis.

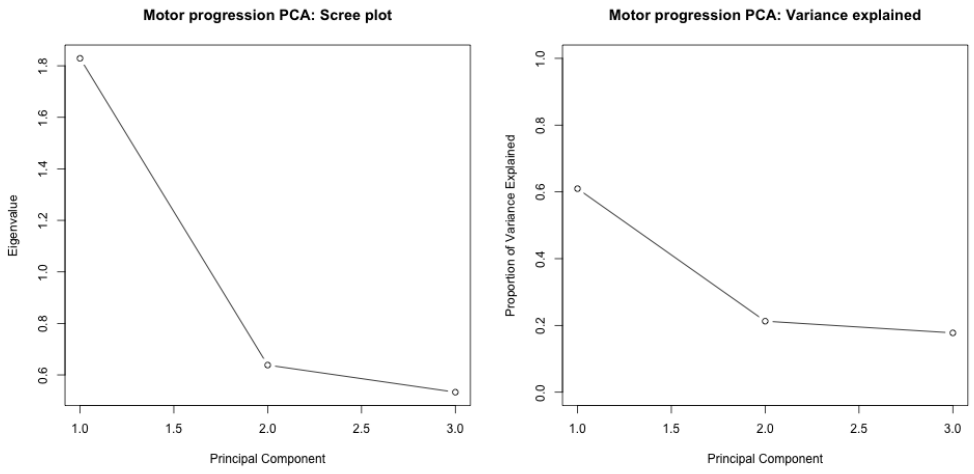

Supplementary Figure 4. Correlation matrix between the random slopes for the motor progression variables.

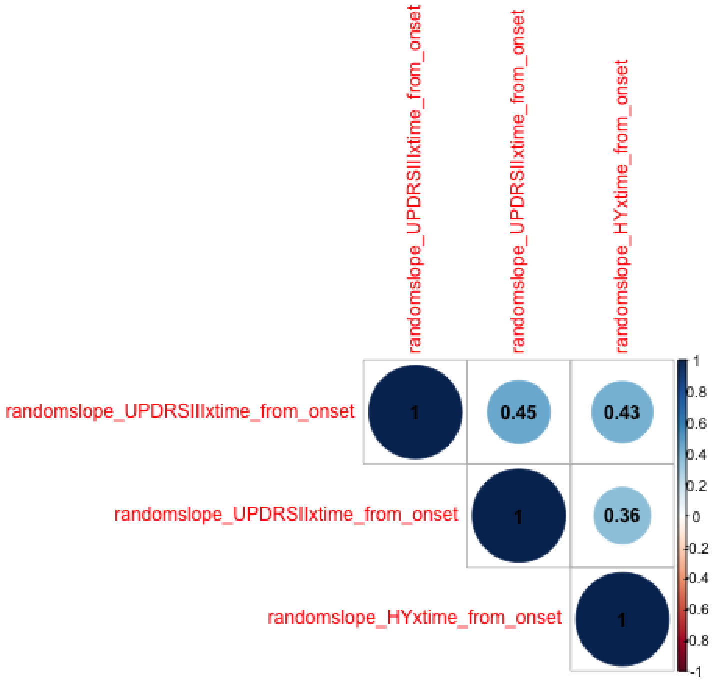

Supplementary Figure 5. Scree plot and plot showing the proportion of variance explained in the cognitive progression principal component analysis.

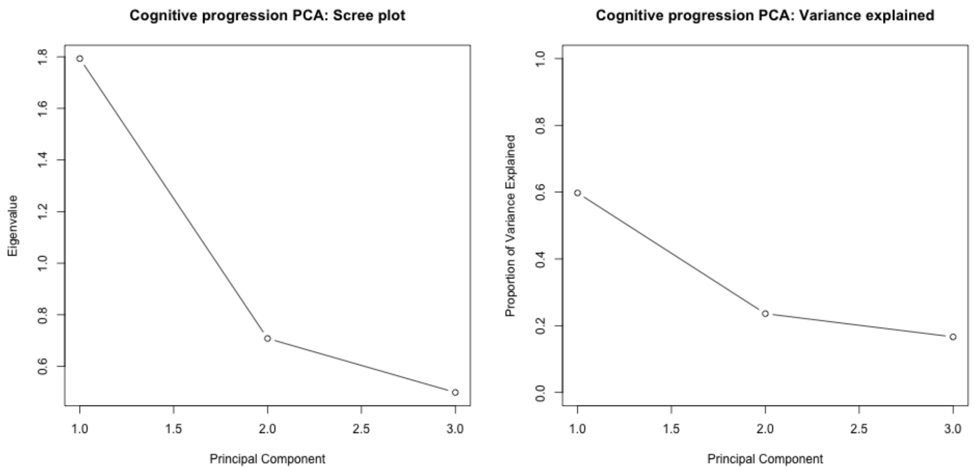

Supplementary Figure 6.Correlation matrix between the random slopes for the cognitive progression variables.

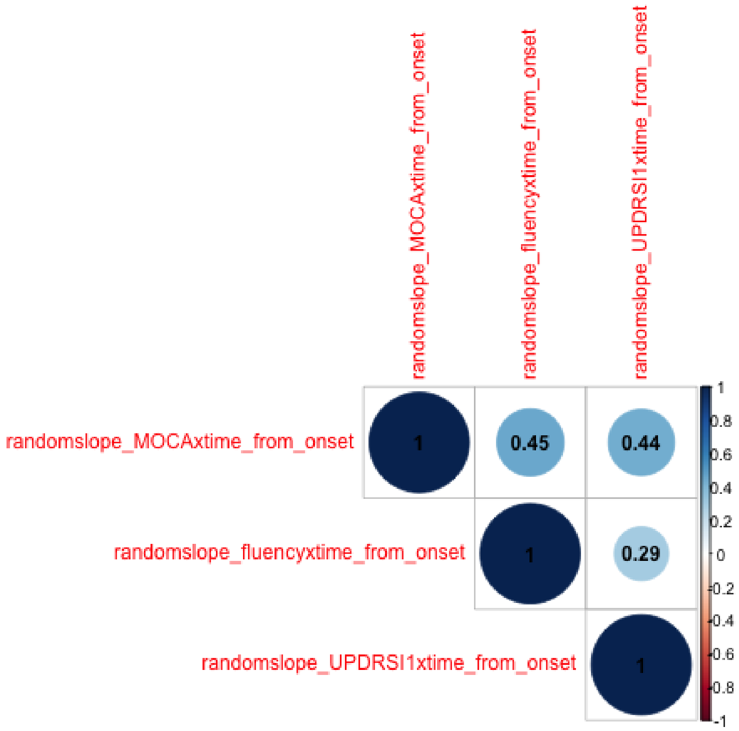

Supplementary Figure 7. Scree plot and plot showing the proportion of variance explained in the composite (cross-domain - both motor and cognitive variables) progression principal component analysis.

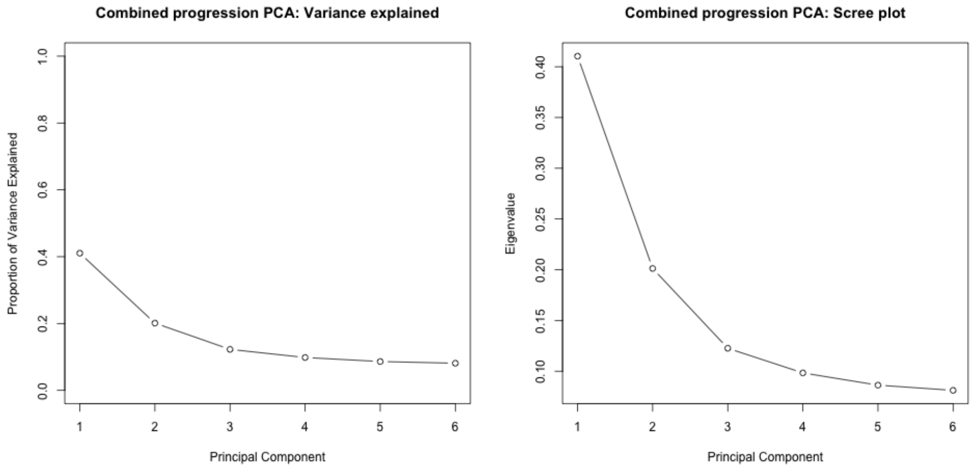

Supplementary Figure 8. Density plot showing the distribution of the composite progression scores, with the normal distribution curve shown in red.

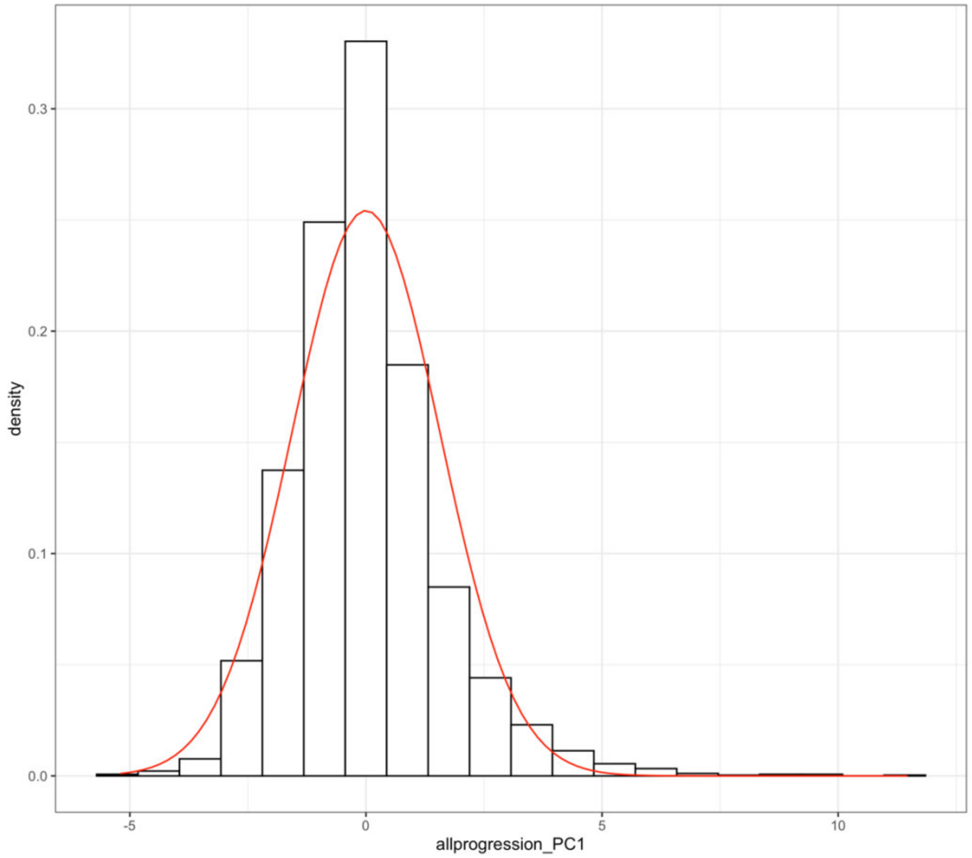

Supplementary Figure 9. Composite progression GWAS: Manhattan plot for the gene-based test from MAGMA.Genome-wide significance indicated by the red dashed line is defined as p = 0.05/17802 (the number of mapped protein coding genes) = 2.81 x 10^{-6}.

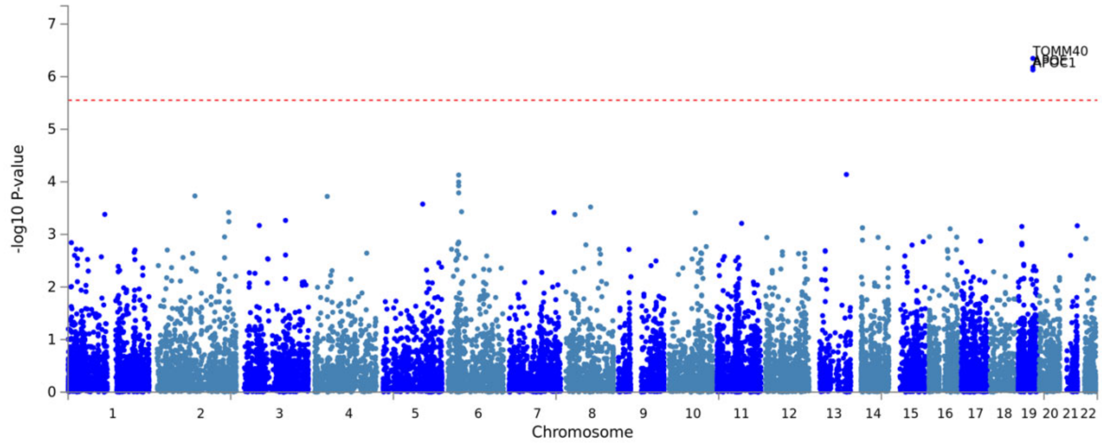

Supplementary Figure 10. QQ plots for the GWAS of composite progression (variant-based and gene-based tests).

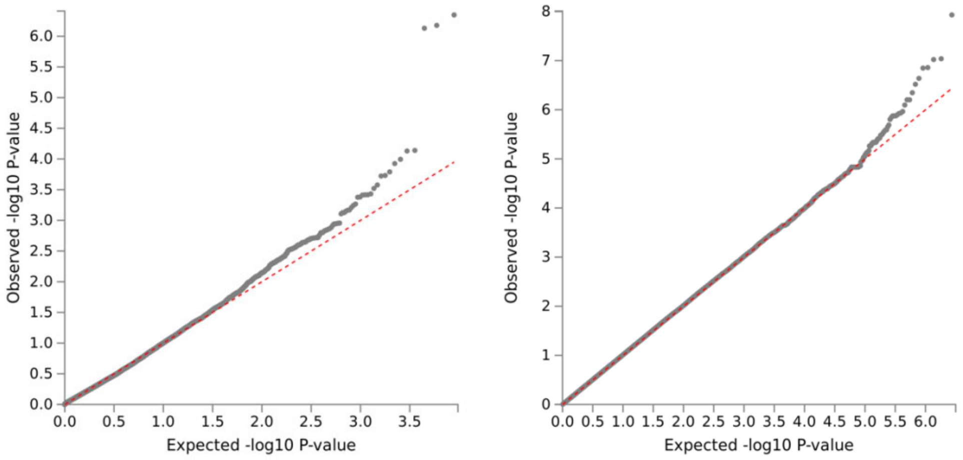

Supplementary Figure 11. Regional association plots of the Chromosome 19 locus associated with composite progression.The recombination rate is shown in the blue line, based on European samples (build GRCh38). Plots were generated using LocusZoom (LocalZoom tool; http://locuszoom.org/).

A) The original GWAS results. The top SNP rs429358 is shown as a purple diamond.

B) Conditioning on the top SNP rs429358. There are no SNPs that are significant after conditioning on rs429358. The SNP shown in purple is rs10414043 (19:44912456).

A

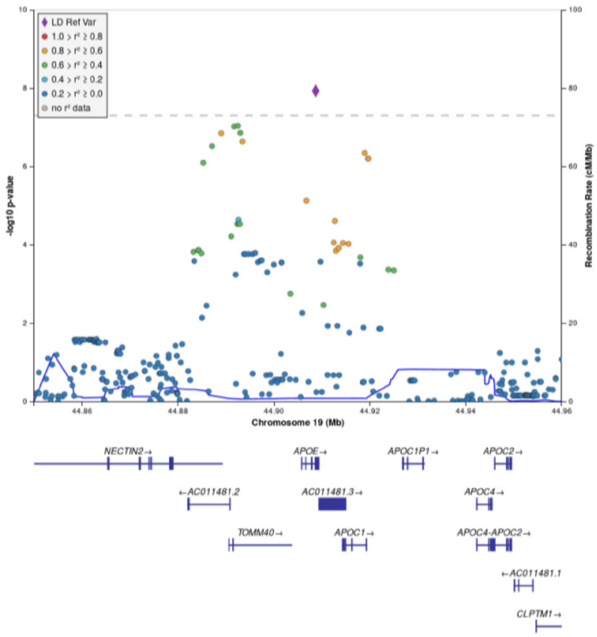

B

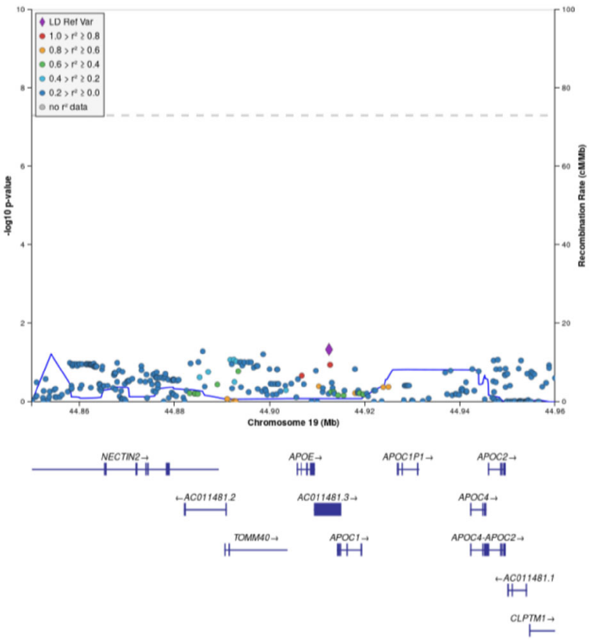

Supplementary Figure 12. Manhattan plot of the gene-based test for motor progression from MAGMA. Genome-wide significance was defined at p = 0.05/17802 (the number of mapped protein coding genes) = 2.81×10^{-6}.

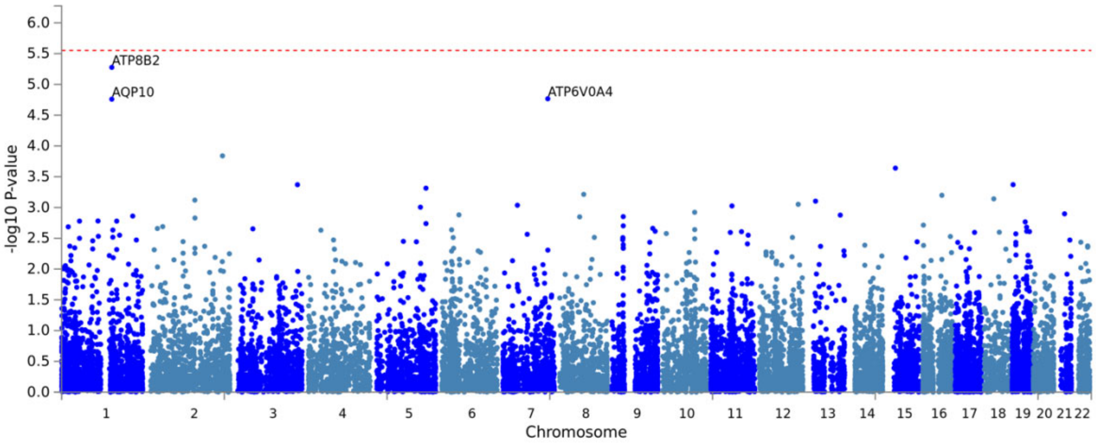

Supplementary Figure 13.QQ plots for the GWAS of motor progression (variant-based and gene-based tests).

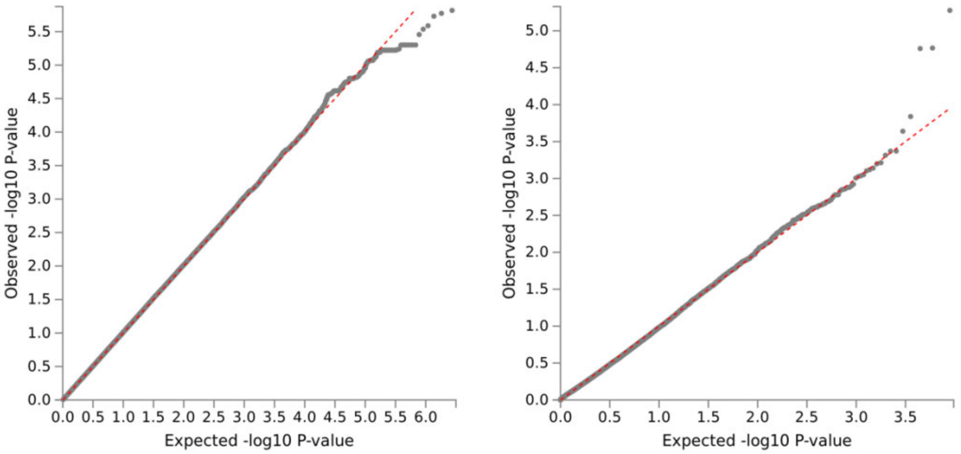

Supplementary Figure 14.Manhattan plot of the gene-based test for cognitive progression from MAGMA. Genome-wide significance was defined at P = 0.05/17802 (the number of mapped protein coding genes) = 2.81 x 10^{-6}.

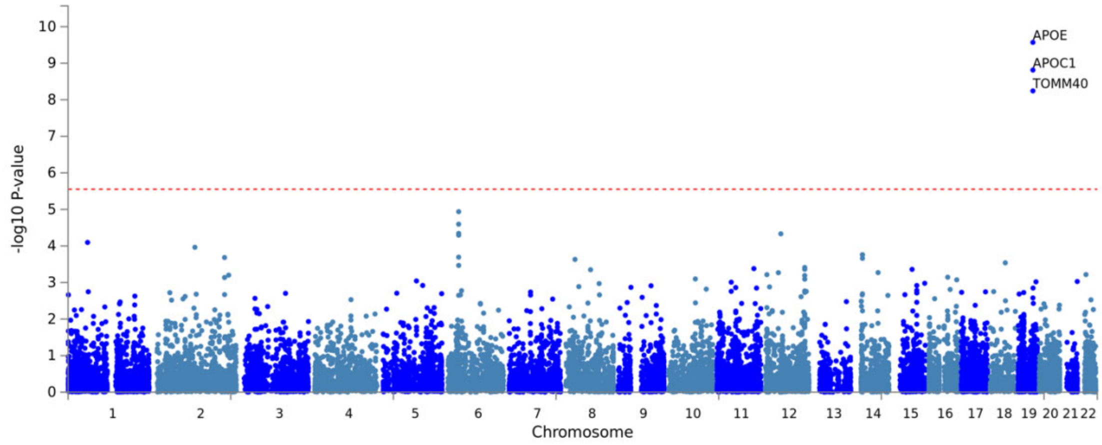

Supplementary Figure 15. QQ plots for the GWAS of cognitive progression (variant-based and gene-based tests).

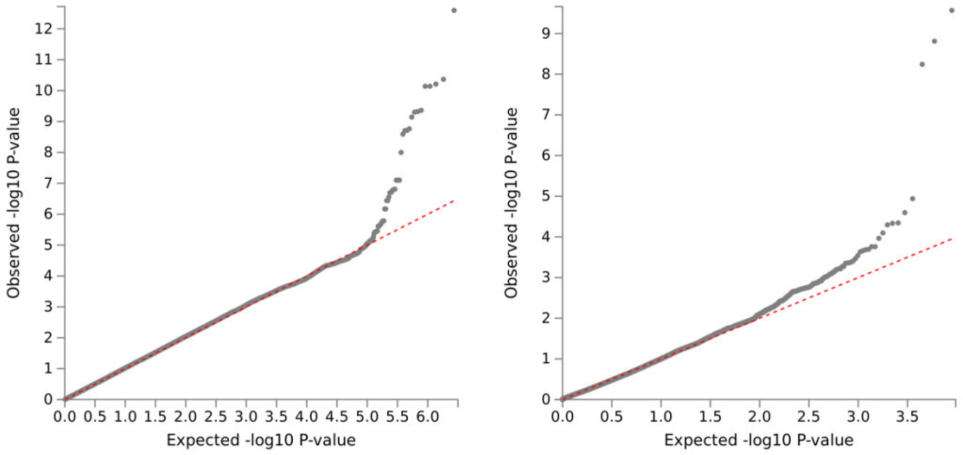

Supplementary Figure 16. Raw MoCA scores in each cohort by *APOE* ε4 status (carriers vs. non-carriers).Note that the cognitive progression score was also based on semantic fluency performance and Part 1.1 of the MDS-UPDRS. Any mean data points with < 5 individuals were removed. Lines show the means ± standard errors of individuals who had data at that timepoint. Some of the means increase over time, likely because of participant drop-out. However, individuals who had data for at least one timepoint were still included in the progression scores and GWAS analysis; this graph is for illustrative purposes and does not capture all the data that was used to create the progression scores. The PPMI cohort were assessed at different timepoints (1 year intervals) than Tracking Parkinson's and Oxford Discovery (1.5 year intervals).

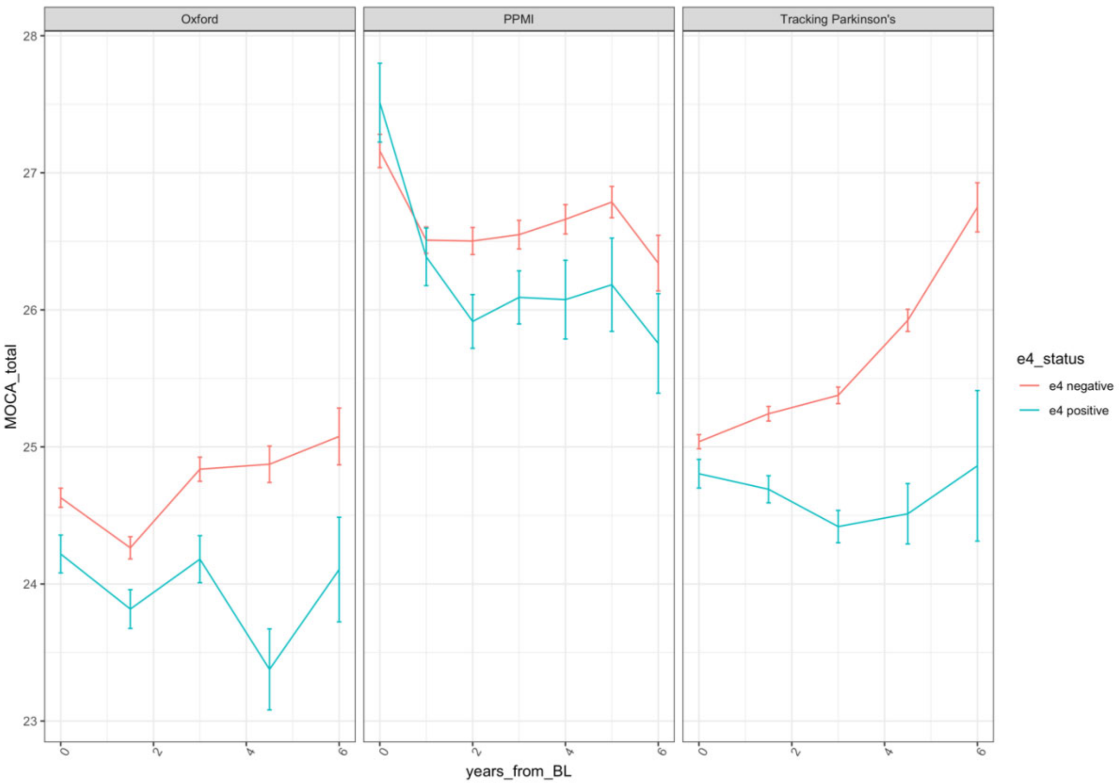

MOCA = Montreal Cognitive Assessment

Supplementary Figure 17. Regional association plots of the Chromosome 19 locus associated with cognitive progression.The recombination rate is shown in the blue line, based on European samples (build GRCh38). Plots were generated using LocusZoom (LocalZoom tool; http://locuszoom.org/). The same region (19:44850000-44960000) is displayed in all plots.

A) The original GWAS results. The top SNP rs429358 is shown as a purple diamond.

B) Conditioning on the top SNP rs429358, revealing a group of SNPs that remain significant after removing the effect associated with rs429358. The top SNP is rs6857.

C) Conditioning on both rs429358 and rs6857, revealing potentially a third independent signal. The top SNP from this conditional analysis is rs12721051.

A: Original cognitive progression GWAS

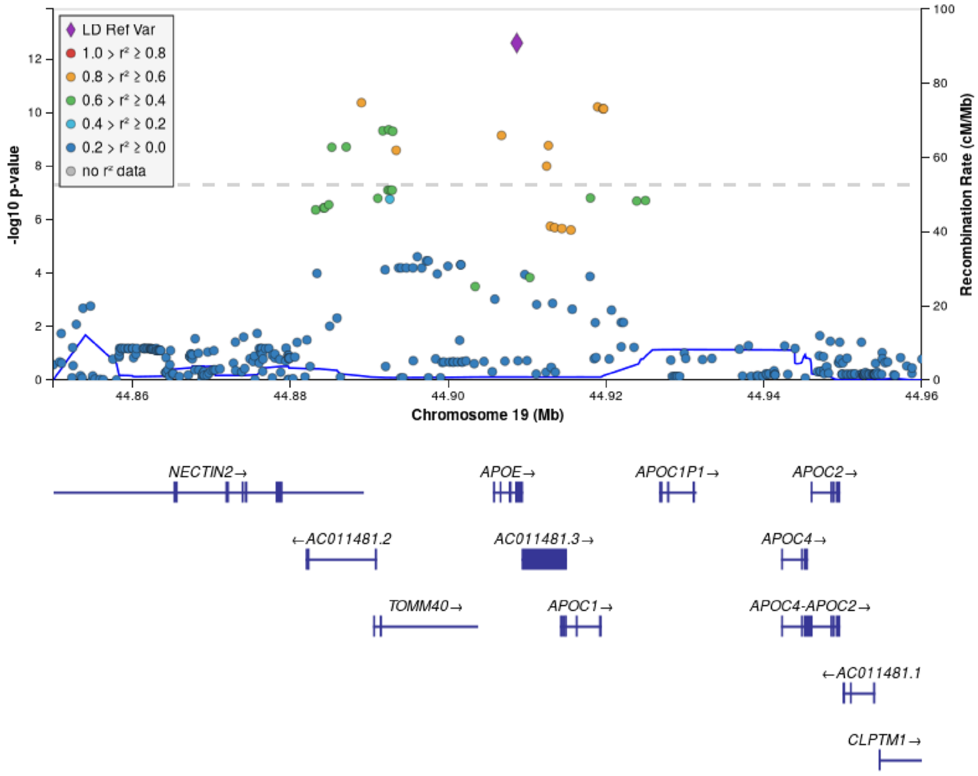

B: Conditioning on rs429358

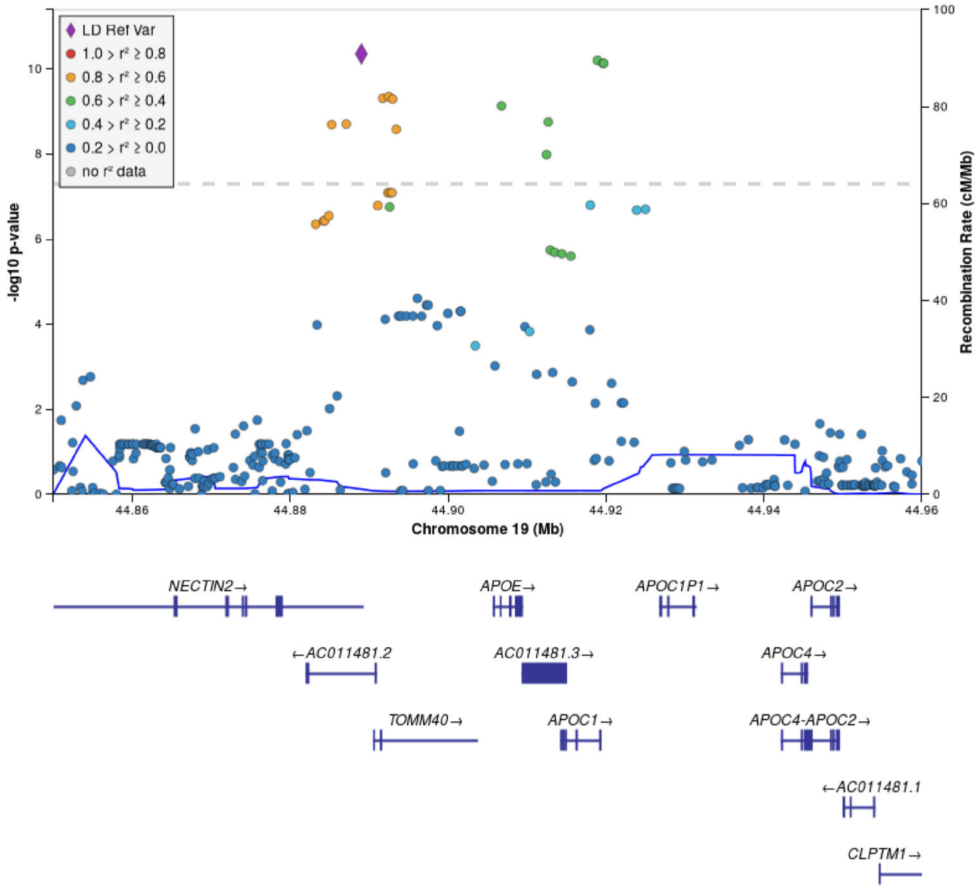

C: Conditioning on rs429358 and rs6857

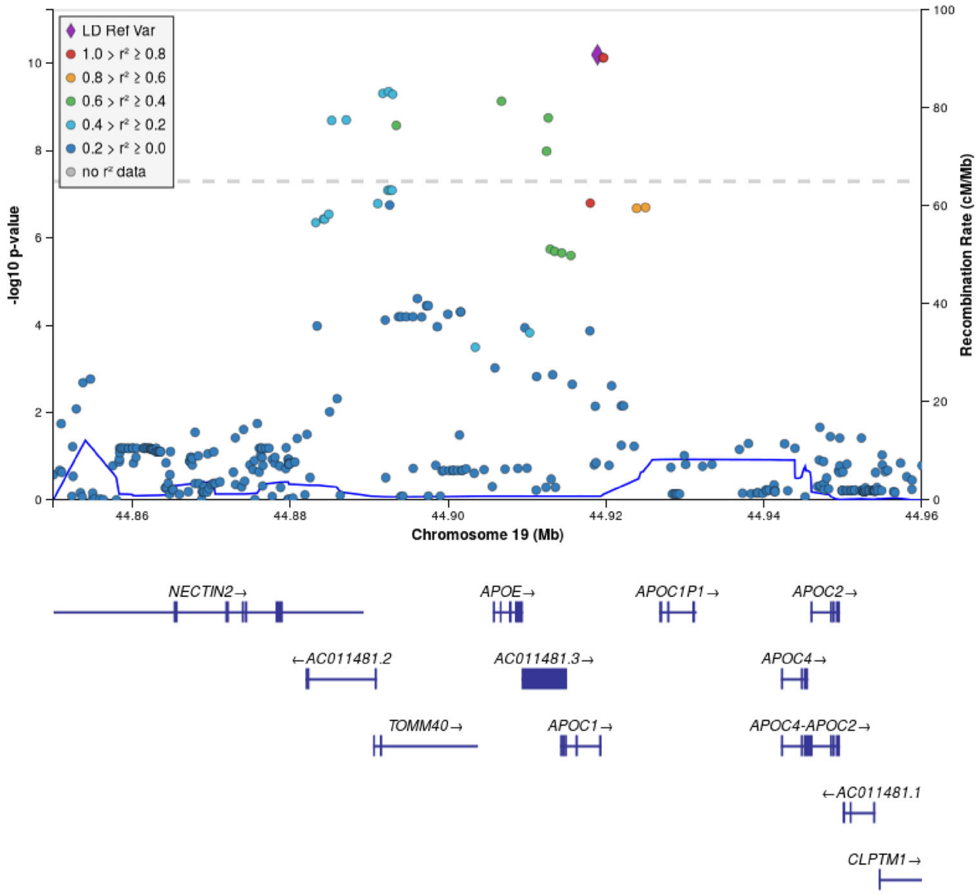

Supplementary Figure 18. Forest plots for the top SNPs for motor and cognitive progression, showing effect sizes of the top SNPs in different cohorts.

A)

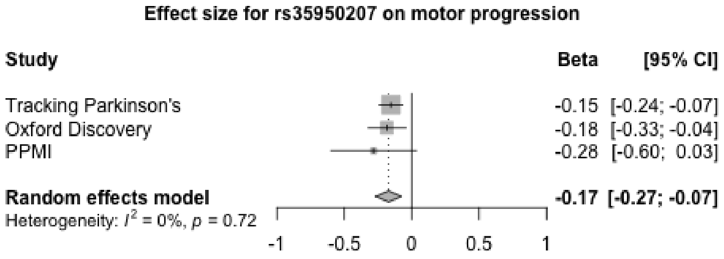

B)

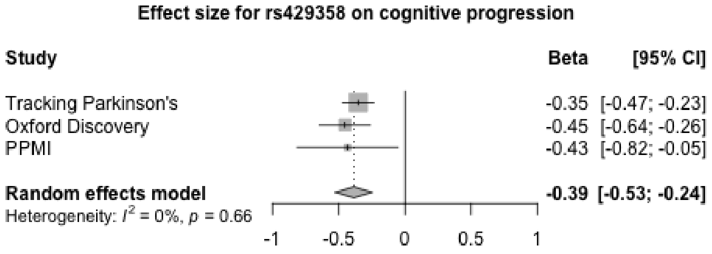

Supplementary Figure 19. Heatmap of the PD GWAS risk loci and their association with composite, motor, or cognitive progression. Only variants with at least one association < 0.05 are shown in the heatmap.

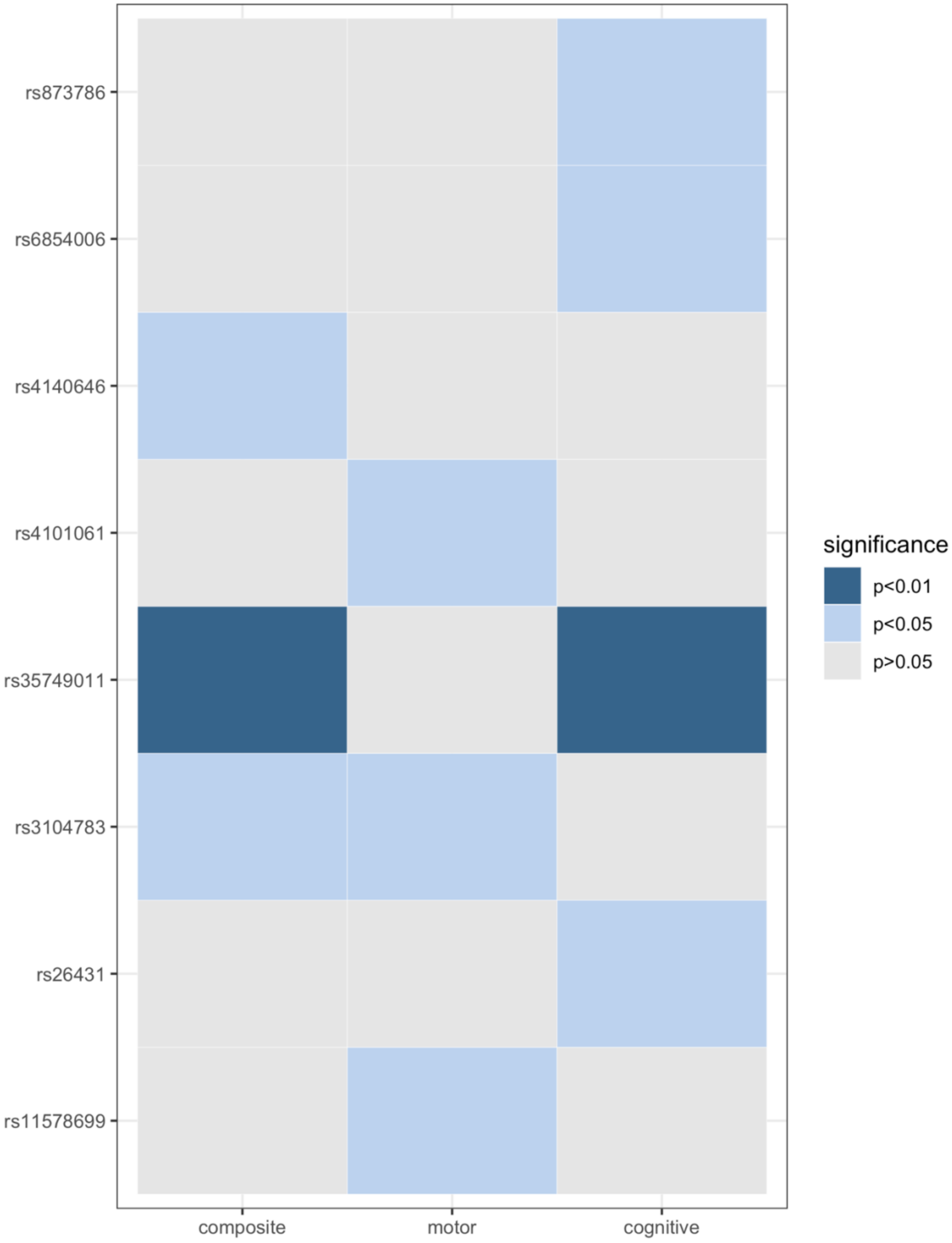

Supplementary Figure 20. Heatmap of candidate variants and their association with composite, motor, or cognitive progression.

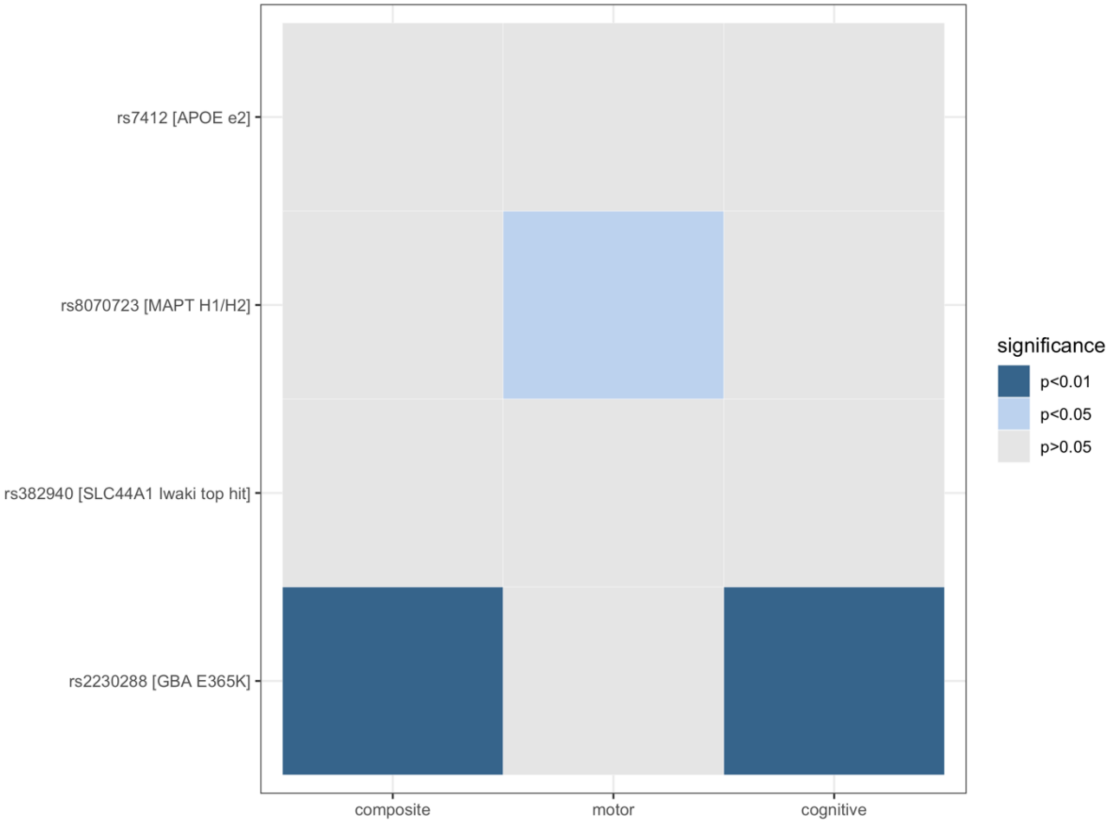

Supplementary Table 1. Correlation between motor progression principal components and cognitive progression principal components. Pearson's r, r2, and p values are reported.

	motor_PC1	motor_PC2	motor_PC3
cog_PC1	$r = -0.35$<br>$r^2 = 0.12$<br>$p < 2.2 \times 10^{-16}$	$r = 0.14$<br>$r^2 = 0.02$<br>$p = 6.2 \times 10^{-15}$	$r = 0.06$<br>$r^2 = 0.004$<br>$p = 4.5 \times 10^{-4}$
cog_PC2	$r = -0.11$<br>$r^2 = 0.01$<br>$p = 1.9 \times 10^{-9}$	$r = 0.09$<br>$r^2 = 0.007$<br>$p = 1.7 \times 10^{-6}$	$r = 0.06$<br>$r^2 = 0.004$<br>$p = 3.0 \times 10^{-4}$
cog_PC3	$r = -0.01$<br>$r^2 = 1.8 \times 10^{-4}$<br>$p = 0.05$	$r = 0.05$<br>$r^2 = 0.003$<br>$p = 3.5 \times 10^{-3}$	$r = 0.005$<br>$r^2 = 2.5 \times 10^{-5}$<br>$p = 0.78$

Supplementary Table 2. Correlation between the principal components from combined progression PCA and random slopes from individual measures. Pearson's r is reported.

	PC1	PC2	PC3	PC4	PC5	PC6
MDS-UPDRSIII	0.65	-0.48	0.12	-0.33	-0.46	0.11
MDS-UPDRSII	0.72	-0.28	-0.29	-0.30	0.40	-0.27
Hoehn and Yahr	0.57	-0.54	0.30	0.51	0.17	0.08
MoCA total	0.67	0.48	0.07	0.22	-0.26	-0.45
Semantic fluency	0.56	0.53	0.50	-0.23	0.22	0.23
MDS-UPDRS 1.1	0.66	0.30	-0.54	0.18	-0.05	0.37
% of variance explained	*41.0%*	*20.1%*	*12.3%*	*9.9%*	*8.6%*	*8.1%*

Supplementary Table 3. Outputs from mixed effects models showing the relationship between the longitudinal change in the raw scales (before percentage transformation and standardisation) and the composite progression score, in each cohort separately.We report the beta coefficients for the fixed effect of the composite progression score PC1 and the marginal R_2. The marginal R_2 shows the proportion of variance in the outcome that is explained by the composite progression score. Note that the scales have not been standardised or transformed, so there are some differences between cohorts, e.g. in the semantic fluency betas because this task was conducted slightly different in each cohort. The smaller beta and R_2 values in the PPMI cohort may be because these patients are slightly slower progressing than the other two cohorts (see main Table 1) relate to their recruitment at an earlier disease stage, younger age, and if they were anticipated not to need PD medication in the following 6 months. Thus the PPMI samples may have less heterogeneity in scale changes and therefore smaller contribution to the PCAs.

	MDS-UPDRS_III	MDS-UPDRS_II	H&Y	MoCA	Semantic fluency	MDS-UPDRS1.1
Tracking Parkinson's	$\beta= 3.62$<br>$R_2 = 0.12$	$\beta = 2.35$<br>$R_2 = 0.15$	$\beta = 0.16$<br>$R_2 = 0.10$	$\beta = -1.17$<br>$R_2 = 0.18$	$\beta = -1.81$<br>$R_2 = 0.13$	$\beta = 0.24$<br>$R_2 = 0.18$
Oxford Discovery	$\beta = 2.82$<br>$R_2 = 0.10$	$\beta = 2.02$<br>$R_2 = 0.16$	$\beta = 0.09$<br>$R_2 = 0.07$	$\beta = -1.15$<br>$R_2 = 0.23$	$\beta = -2.43$<br>$R_2 = 0.15$	$\beta = 0.22$<br>$R_2 = 0.21$
PPMI	$\beta = 1.05$<br>$R_2 = 0.03$	$\beta = 0.76$<br>$R_2 = 0.07$	$\beta = 0.05$<br>$R_2 = 0.04$	$\beta = -0.31$<br>$R_2 = 0.04$	$\beta = -0.75$<br>$R_2 = 0.09$	$\beta = 0.11$<br>$R_2 = 0.11$

Supplementary Table 4. Correlations between the composite progression components and the motor and cognitive components. Pearson's r is reported.

	motor_PC1	motor_PC2	motor_PC3	cog_PC1	cog_PC2	cog_PC3
allprogression_PC1	0.83	-0.11	-0.05	-0.82	-0.10	-0.01
allprogression_PC2	-0.55	-0.23	-0.08	-0.57	0.19	0.01
allprogression_PC3	0.05	0.51	0.19	-0.02	0.87	0.07
allprogression_PC4	-0.06	0.76	-0.39	-0.08	-0.35	0.27
allprogression_PC5	0.03	-0.12	-0.85	0.05	0.22	-0.40
allprogression_PC6	-0.03	0.27	0.28	-0.04	-0.14	-0.87

Supplementary Table 5. Correlations between the motor principal components and the random slopes from the individual motor measures.

	motor_PC1	motor_PC2	motor_PC3
MDS-UPDRSIII	0.81	-0.07	0.58
MDS-UPDRSII	0.77	-0.52	-0.37
Hoehn and Yahr	0.76	0.61	-0.24

Supplementary Table 6. Correlations between the cognitive principal components and the random slopes from the individual motor measures.

	cog_PC1	cog_PC2	cog_PC3
MoCA	-0.83	0.02	0.55
Semantic fluency	-0.75	0.58	-0.33
MDS-UPDRS 1.1	-0.74	-0.61	-0.29

Supplementary Table 7. Top 10 independent SNPs from the GWAS of composite progression.

Chr	Position (GRCh38)	SNP	Effect allele (minor)	Ref allele	Effect allele freq	Nearest gene	Distance to gene (kb)	Beta	SE	p value original	p value conditional (COJO)
19	44908684	rs429358	C	T	0.14	APOE	0	0.35	0.06	1.17E-08	1.07E-08
10	33942102	rs224750	T	C	0.37	PARD3	167458	-0.21	0.04	1.09E-06	1.20E-06
15	94318611	rs11634227	C	T	0.41	MCTP2	0	-0.21	0.04	1.19E-06	1.32E-06
19	50760039	rs4802739	C	A	0.40	GPR32	10425	0.20	0.04	1.27E-06	1.05E-06
6	119112570	rs79987229	T	A	0.01	FAM184A	0	0.85	0.18	2.57E-06	1.21E-06
15	45744252	rs17554587	C	G	0.22	SQRDL	52958	0.24	0.05	3.11E-06	3.39E-06
5	4699328	rs62343939	T	C	0.05	ADAMTS16	441002	0.43	0.09	3.25E-06	3.52E-06
5	122191027	rs17367669	T	G	0.22	LOC100505841	8364	0.23	0.05	3.31E-06	3.59E-06
7	17673826	rs10253857	T	C	0.22	SNX13	116935	-0.23	0.05	3.86E-06	4.19E-06
2	108292945	rs13424530	A	G	0.44	SULT1C2	0	0.20	0.04	4.06E-06	3.25E-06

Supplementary Table 8. Top 10 independent SNPs from the GWAS of motor progression.

Chr	Position (GRCh38)	SNP	Effect allele (minor)	Ref allele	Effect allele freq	Nearest gene	Distance to gene (kb)	Beta	SE	p value original	p value conditional
5	122193658	rs5870994	C	CTT	0.23	LOC100505841	10995	0.21	0.04	1.36E-06	1.49E-06
9	8454921	rs7870456	T	C	0.22	PTPRD	0	0.21	0.04	1.53E-06	1.68E-06
15	94320087	rs72767442	A	T	0.41	MCTP2	0	-0.18	0.04	1.69E-06	1.85E-06
2	23493673	rs6741991	G	A	0.26	KLHL29	0	0.20	0.04	2.91E-06	3.17E-06
1	154319482	rs35950207	T	C	0.31	AQP10	1585	-0.18	0.04	5.01E-06	5.40E-06
6	119067987		T	TAAAC	0.01	FAM184A	0	0.70	0.15	5.03E-06	5.40E-06
12	5829410	rs74709761	C	G	0.04	ANO2	0	-0.41	0.09	6.42E-06	8.72E-06
11	114821560	rs4436579	T	C	0.29	NXPE2	114443	0.18	0.04	7.47E-06	8.02E-06
12	12677103	rs12813102	C	A	0.04	GPR19	0	0.43	0.10	7.70E-06	1.05E-05
15	71520619	rs4128840	A	G	0.41	THSD4	0	-0.17	0.04	7.95E-06	8.53E-06

Supplementary Table 9. Motor progression GWAS performed in each cohort separately. Progression scores were created in the merged cohort. The results for the top 5 independent hits from the combined motor progression GWAS are shown here. These show that the effects and allele frequencies are consistent across all three cohorts.

**SNP**	**Nearest gene**	**Combined**			**Tracking Parkinson's**			**Oxford**			**PPMI**		
		Beta	*p*	*MAF*	*Beta*	*p*	*MAF*	*Beta*	*p*	*MAF*	*Beta*	*p*	*MAF*
rs5870994	LOC100505841	0.21	1.36E-06	0.23	0.20	5.31e-05	0.23	0.17	0.04	0.21	0.30	0.064	0.26
rs7870456	PTPRD	0.21	1.53E-06	0.22	0.12	0.016	0.23	0.32	3.81e-05	0.23	0.36	0.052	0.21
rs72767442	MCTP2	-0.18	1.69E-06	0.41	-0.14	0.001	0.41	-0.15	0.026	0.40	-0.40	0.005	0.41
rs6741991	KLHL29	0.20	2.91E-06	0.26	0.10	0.040	0.25	0.17	0.022	0.26	0.65	7.19e-05	0.27
rs35950207	AQP10	-0.18	5.01E-06	0.31	-0.15	0.0007	0.31	-0.18	0.010	0.30	-0.28	0.080	0.32

Supplementary Table 10. Motor progression GWAS performed for each scale separately. The results for the top 5 independent hits from the combined motor progression GWAS are shown here. The random slope from the mixed effects model for each scale was used as the progression measure. These results show that the effects are consistent across each of the different motor scales.

**SNP**	**Nearest gene**	**Combined**		**MDS-UPDRSIII random slope**		**MDS-UPDRSII random slope**		**Hoehn and Yahr random slope**	
		Beta	*p*	*Beta*	*p*	*Beta*	*p*	*Beta*	*p*
rs5870994	LOC100505841	0.21	1.36E-06	0.013	2.32e-05	0.012	9.43e-05	0.006	0.0008
rs7870456	PTPRD	0.21	1.53E-06	0.008	0.008	0.008	0.012	0.010	4.74e-10
rs72767442	MCTP2	-0.18	1.69E-06	-0.013	1.42e-06	-0.008	0.003	-0.004	0.006
rs6741991	KLHL29	0.20	2.91E-06	0.011	0.0004	0.010	0.002	0.007	1.08e-05
rs35950207	AQP10	-0.18	5.01E-06	-0.014	3.21e-06	-0.005	0.106	-0.006	2.79e-05

Supplementary Table 11. Top 10 independent SNPs from the GWAS of cognitive progression.

Chr	Position (GRCh38)	SNP	Effect allele (minor)	Ref allele	Effect allele freq	Nearest gene	Distance to gene (kb)	Beta	SE	p value original	p value condition al
19	44908684	rs429358	C	T	0.14	APOE	0	-0.38	0.05	2.53E-13	4.20E-13
12	20812884	rs143371462	G	A	0.02	SLCO1B3	0	-0.64	0.13	6.76E-07	7.53E-07
3	23951314	rs113730632	G	A	0.05	NR1D2	0	0.41	0.09	1.65E-06	6.59E-07
12	125083207	rs6488987	C	T	0.36	AACS	0	0.18	0.04	1.65E-06	1.95E-06
11	8882396	rs34105455	G	A	0.13	ST5	0	-0.25	0.05	3.64E-06	3.94E-06
8	74970819	rs2956605	A	C	0.40	CRISPLD1	13654	-0.17	0.04	3.70E-06	4.02E-06
11	107127349	rs17092224	C	G	0.11	CWF19L2	198996	-0.27	0.06	3.85E-06	4.62E-06
22	34970241	rs5755468	C	T	0.37	ISX-AS1	0	-0.17	0.04	4.16E-06	4.49E-06
9	84627637	rs148603475	T	C	0.08	NTRK2	40821	-0.31	0.07	6.08E-06	8.94E-06
20	18097908	rs1124933	A	G	0.42	PET117	39947	-0.16	0.04	7.94E-06	8.50E-06

Supplementary Table 12. Cognitive progression GWAS performed in each cohort separately. Progression scores were created in the merged cohort. The results for the top 5 independent hits from the combined cognitive progression GWAS are shown here. These show that the effects and allele frequencies are consistent across all three cohorts.

SNP	Nearest gene	Combined			Tracking Parkinson's			Oxford			PPMI		
		Beta	*p*	*MAF*	*Beta*	*p*	*MAF*	*Beta*	*p*	*MAF*	*Beta*	*p*	*MAF*
rs429358	APOE	-0.38	2.53E-13	0.14	-0.35	6.88e-09	0.14	-0.45	3.56e-06	0.14	-0.43	0.026	0.13
rs143371462	SLCO1B3	-0.64	6.76E-07	0.02	-0.54	0.0002	0.02	-0.73	0.003	0.02	-1.04	0.049	0.02
rs113730632	NR1D2	0.41	1.65E-06	0.05	0.36	0.0004	0.05	0.19	0.240	0.05	0.80	0.004	0.06
rs6488987	AACS	0.18	1.65E-06	0.36	0.21	1.66e-06	0.37	0.05	0.428	0.36	0.29	0.042	0.34
rs34105455	ST5	-0.25	3.64E-06	0.13	-0.15	0.018	0.13	0.10	0.0002	0.12	-0.39	0.047	0.14

Supplementary Table 13. Cognitive progression GWAS performed for each scale separately.The results for the top 5 independent hits from the combined cognitive progression GWAS are shown here. The random slope from the mixed effects model for each scale was used as the progression measure. These results show that the direction of effects and p values are consistent across each of the different cognitive scales.

SNP	Nearest gene	Combined		MoCA random slope		Fluency random slope		MDS-UPDRS 1.1 random slope	
		Beta	*p*	*Beta*	*p*	*Beta*	*p*	*Beta*	*p*
rs429358	APOE	-0.38	2.53E-13	0.02	6.84e-13	0.007	4.04e-06	0.02	1.04e-07
rs143371462	SLCO1B3	-0.64	6.76E-07	0.03	0.0001	0.02	0.0001	0.03	9.78e-05
rs113730632	NR1D2	0.41	1.65E-06	-0.02	0.0004	-0.01	1.595e-05	-0.01	0.006
rs6488987	AACS	0.18	1.65E-06	-0.007	0.002	-0.004	9.16e-05	-0.009	2.37e-05
rs34105455	ST5	-0.25	3.64E-06	0.01	2.64e-05	0.006	0.0008	0.009	0.002

Supplementary Table 14. APOE genotype frequencies.Percentages are shown of the total number of patients (N = 2949) who had genotyping data available after quality control and removal of population outliers.

***APOE* genotype**	**N**	**% of total (N = 2949)**
e2/e2	15	0.5
e2/e3	385	13.1
e2/e4	87	3.0
e3/e3	1783	60.5
e3/e4	624	21.2
e4/e4	55	1.9
e2 negative	2462	83.49
e2 positive	487	16.51
e4 negative	2183	74.03
e4 positive	766	25.97

Supplementary Table 15. PD risk SNPs missing for calculation of the genetic risk score, and whether a proxy was identified. All other SNPs from Nalls et al. (2019) were present in our final genotype dataset. In total, 75 SNPs were used for creation of the genetic risk score including 2 proxies.

SNP	chr_pos_GRCh38	Effect allele	Other allele	EAF	Proxy identified	R_2 for proxy	D' for proxy	Correlated alleles
rs114138760	1:154925709	c	g	0.0112	No			
rs76763715	1:155235843	t	c	0.9953	No			
rs6658353	1:161499264	c	g	0.5011	No			
rs73038319	3:18320267	a	c	0.9592	No			
rs13117519	4:113447909	t	c	0.1744	No			
rs75859381	6:132889222	t	c	0.9673	No			
rs76949143	7:66544864	a	t	0.0507	rs62469072	0.9717	1	T=C,A=T
rs117896735	10:119776815	a	g	0.0166	No			
rs34637584	12:40340400	a	g	0.0015	No			
rs10847864	12:122842051	t	g	0.364	No			
rs11610045	12:132487182	a	g	0.4896	No			
rs6497339	16:19266171	a	t	0.4536	No			
rs62053943	17:45666837	t	c	0.1552	No			
rs117615688	17:45720942	a	g	0.067	No			
rs11658976	17:46789439	a	g	0.5802	No			
rs61169879	17:61840005	t	c	0.1641	rs3744434	0.935	0.9777	C=A,T=C

Supplementary Table 16. GBA variants included as pathogenic, and their frequencies.Note that some individuals carried more than one variant. Frequencies are shown as a percentage of the total number of patients in Tracking Parkinson’s and PPMI who were screened for GBA with sequencing (N= 2020).

Variant	Number of carriers (%)
p.E326K	103 (5.1%)
p.L444P	25 (1.2%)
p.N370S	14 (0.7%)
p.T369M	46 (2.3%)
p.G202R	2 (0.10%)
p.R463C	6 (0.30%)
p.D409H	1 (0.05%)
p.F213I	1 (0.05%)
p.G377S	1 (0.05%)
p.R257Q	1 (0.05%)

Supplementary Table 17. Sensitivity analysis excluding PD cases with less than 90% diagnostic certainty. The top SNPs in the main analysis are shown, with the results from the sensitivity analysis for comparison. 5.2% (51/985) patients were removed from Oxford Discovery, 21.3% (419/1966) patients were removed from Tracking Parkinson's.

**SNP**	**Nearest gene**	**Results for top SNPs in full dataset**		**Results in PD cases with ≥ 90% diagnostic certainty**		
		Beta	*p*	*Beta*	*p*	*N*
**Composite progression**						
rs429358	APOE	0.35	1.17E-08	0.34	6.00e-07	2459
rs224750	PARD3	-0.21	1.09E-06	-0.20	5.36e-05	2459
rs11634227	MCTP2	-0.21	1.19E-06	-0.20	2.14e-05	2459
rs4802739	GPR32	0.20	1.27E-06	0.22	2.86e-06	2459
rs79987229	FAM184A	0.85	2.57E-06	0.99	3.64e-07	2459
**Motor progression**						
rs5870994	LOC100505841	0.21	1.36E-06	0.19	4.19e-05	2496
rs7870456	PTPRD	0.21	1.53E-06	0.19	0.0001	2496
rs72767442	MCTP2	-0.18	1.69E-06	-0.15	0.0001	2496
rs6741991	KLHL29	0.20	2.91E-06	0.19	2.27e-05	2496
rs35950207	AQP10	-0.18	5.01E-06	-0.18	2.13e-05	2496
**Cognitive progression**						
rs429358	APOE	-0.38	2.53E-13	-0.39	2.05e-12	2474
rs143371462	SLCO1B3	-0.64	6.76E-07	-0.64	3.22e-06	2474
rs113730632	NR1D2	0.41	1.65E-06	0.43	4.34e-06	2474
rs6488987	AACS	0.18	1.65E-06	0.19	1.69e-06	2474
rs34105455	ST5	-0.25	3.64E-06	-0.25	1.79e-05	2474

Supplementary Table 18. Sensitivity analysis excluding fastest and slowest progressing cases (top and bottom 5% of each distribution)

**SNP**	**Nearest gene**	**Results for top SNPs in full dataset**		**Results in PD cases excluding extreme 5%**		
		Beta [95% CI]	*p*	*Beta*	*p*	*N*
**Composite progression**						
rs429358	APOE	0.35 [0.23, 0.47]	1.17E-08	0.17	0.0002	2483
rs224750	PARD3	-0.21 [-0.30, -0.13]	1.09E-06	-0.10	0.002	2483
rs11634227	MCTP2	-0.21 [-0.29, -0.12]	1.19E-06	-0.10	0.003	2483
rs4802739	GPR32	0.20 [0.12, 0.29]	1.27E-06	0.11	0.004	2483
rs79987229	FAM184A	0.85 [0.50, 1.21]	2.57E-06	0.42	0.002	2483
**Motor progression**						
rs5870994	LOC100505841	0.21 [0.12, 0.29]	1.36E-06	0.12	0.0003	2570
rs7870456	PTPRD	0.21 [0.12, 0.29]	1.53E-06	0.10	0.002	2570
rs72767442	MCTP2	-0.18 [-0.25, -0.10]	1.69E-06	-0.07	0.012	2570
rs6741991	KLHL29	0.20 [0.11, 0.28]	2.91E-06	0.08	0.008	2570
rs35950207	AQP10	-0.18 [-0.26, -0.10]	5.01E-06	-0.06	0.029	2570
**Cognitive progression**						
rs429358	APOE	-0.38 [-0.48, -0.28]	2.53E-13	-0.17	1.28e-05	2511
rs143371462	SLCO1B3	-0.64 [-0.89, -0.39]	6.76E-07	-0.32	0.001	2511
rs113730632	NR1D2	0.41 [0.24, 0.58]	1.65E-06	0.17	0.006	2511
rs6488987	AACS	0.18 [0.11, 0.25]	1.65E-06	0.09	0.002	2511
rs34105455	ST5	-0.25 [-0.35, -0.14]	3.64E-06	-0.15	0.0002	2511

References

1. Malek N, Swallow DMA, Grosset KA, et al. Tracking Parkinson's: Study Design and Baseline Patient Data. *J Parkinsons Dis*. 2015;5:947-959. doi:10.3233/JAD-2012-120751

2. Szewczyk-Krolikowski K, Tomlinson P, Nithi K, et al. The influence of age and gender on motor and non-motor features of early Parkinson's disease: Initial findings from the Oxford Parkinson Disease Center (OPDC) discovery cohort. *Park Relat Disord*. 2014;20(1):99-105. doi:10.1016/j.parkreldis.2013.09.025

3. Marek K, Jennings D, Lasch S, et al. The Parkinson Progression Marker Initiative (PPMI). *Prog Neurobiol*. 2011;95(4):629-635. doi:10.1016/j.pneurobio.2011.09.005

4. The 1000 Genomes Project Consortium. A global reference for human genetic variation. *Nature*. 2015;526(7571):68-74. doi:10.1038/nature15393

5. Watanabe K, Taskesen E, Van Bochoven A, Posthuma D. Functional mapping and annotation of genetic associations with FUMA. *Nat Commun*. 2017;8(1):1-10. doi:10.1038/s41467-017-01261-5

6. de Leeuw CA, Mooij JM, Heskes T, Posthuma D. MAGMA: Generalized Gene-Set Analysis of GWAS Data. *PLoS Comput Biol*. 2015;11(4):1-19. doi:10.1371/journal.pcbi.1004219

7. Bulik-Sullivan B, Loh PR, Finucane HK, et al. LD score regression distinguishes confounding from polygenicity in genome-wide association studies. *Nat Genet*. 2015;47(3):291-295. doi:10.1038/ng.3211

8. Bulik-Sullivan B, Finucane HK, Anttila V, et al. An atlas of genetic correlations across human diseases and traits. *Nat Genet*. 2015;47(11):1236-1241. doi:10.1038/ng.3406

9. Nalls MA, Blauwendraat C, Vallerga CL, et al. Identification of novel risk loci, causal insights, and heritable risk for Parkinson's disease: a meta-analysis of genome-wide association studies. *Lancet Neurol*. 2019;18(12):1091-1102. doi:10.1016/S1474-4422(19)30320-5

10. Tobin MD, Sheehan NA, Scurrah KJ, Burton PR. Adjusting for treatment effects in studies of quantitative traits: Antihypertensive therapy and systolic blood pressure. *Stat Med*. 2005;24(19):2911-2935. doi:10.1002/sim.2165

11. Fahn S, Oakes D, Shoulson I, et al. Levodopa and the progression of Parkinson's disease. *N Engl J Med*. 2004;351(24):2498-2508. doi:10.1056/NEJMoa033447

12. Goetz CG, Stebbins GT, Tilley BC. Calibration of unified Parkinson's disease rating scale scores to Movement Disorder Society-unified Parkinson's disease rating scale scores. *Mov Disord*. 2012;27(10):1239-1242. doi:10.1002/mds.25122

13. Lawton M, Baig F, Toulson G, et al. Blood biomarkers with Parkinson's disease clusters and prognosis: the Oxford Discovery cohort. *Mov Disord*. 2019;1:1-9. doi:10.1002/mds.27888
